# Venous and arterial thromboembolic events after COVID-19 during the Omicron period in three European countries

**DOI:** 10.1038/s41598-026-51445-7

**Published:** 2026-05-15

**Authors:** Xintong Li, Annika M. Jödicke, Albert Prats-Uribe, Antonella Delmestri, Katia Verhamme, Mees Mosseveld, James T. Brash, Dina Vojinovic, Anna Palomar-Cros, Laura Pérez Crespo, Talita Duarte-Salles, Marek Oja, Raivo Kolde, Edward Burn, Denise Umuhire, Daniel R. Morales, Martí Català

**Affiliations:** 1 Health Data Sciences, Botnar Research Centre, University of Oxford, Oxford, United Kingdom; 2 Department of Medical Informatics, Erasmus University Medical Center, Rotterdam, The Netherlands; 3 IQVIA, Real world Solutions, London, United Kingdom; 4 IQVIA, Real World Solutions, Amsterdam, The Netherlands; 5 Fundació Institut Universitari per a la recerca a l’Atenció Primària de Salut Jordi Gol i Gurina (IDIAPJGol), Barcelona, Spain; 6 Institute of Computer Science, University of Tartu, Tartu, Estonia; 7 European Medicines Agency, Amsterdam, The Netherlands; 8 Usher Institute, University of Edinburgh, Edinburgh, United Kingdom

**Keywords:** Diseases, Health care, Medical research, Microbiology

## Abstract

Patients with earlier SARS-CoV-2 variants are at increased risk of venous and arterial thromboembolic (VTE, ATE) events. Here we aimed to contextualise the incidence of thromboembolic events among patients with COVID-19 during the Omicron period. We conducted a population-based cohort study using electronic health records from the UK (CPRD GOLD), the Netherlands (IPCI), and Spain (SIDIAP) within the DARWIN EU^®^ network. Two cohorts were included: a pre-pandemic population (2017–2019) and individuals infected with SARS-CoV-2 during the Omicron-dominant period. We estimated incidence rates (IRs) of VTE, ATE, and other cardiovascular events at 30-, 60-, 90-, and 180-days post-infection. Crude incidence rate ratios (IRRs) and age-sex standardized incidence ratios (SIRs) were calculated relative to the pre-pandemic cohort. Analyses were stratified by prior infection, vaccination status, and immunocompromised status. In total, we included over 7.6 million individuals (CPRD GOLD: 5.28 M; IPCI: 1.59 M; SIDIAP: 0.75 M) in the general population cohort, and about 0.8 million individuals (CPRD GOLD: 248,847; IPCI: 330,200; SIDIAP: 200,563) in the COVID-19 Omicron cohort. Crude IRs varied by outcome and data source. For VTE, IRs per 100,000 person-years were 136 [95%CI 131–141] in SIDIAP, 167 [164–169] in CPRD GOLD, and 264 [259–270] in IPCI. Elevated SIRs for VTE and ATE were observed following SARS-CoV-2 infection, highest within 30 days and persisting up to 180 days. In CPRD GOLD, the VTE SIR was 3.61 [2.45–5.53] at 30 days, decreasing to 1.88 [1.52–2.34] at 180 days. Higher SIRs were observed among immunocompromised individuals and those without prior infection. Our findings indicate that among individuals diagnosed with SARS-CoV-2 infection during the Omicron-dominant period, observed rates of thromboembolic events exceeded expected background incidence, particularly in the early post-infection period.

**Supplementary Information:**

The online version contains supplementary material available at10.1038/s41598-026-51445-7.

## Introduction

Coronavirus disease-2019 (COVID-19), caused by SARS-CoV-2 infection, has been associated with an increased risk for venous thromboembolism and arterial thromboembolic (VTE, ATE) events^1–3^. Studies assessing the incidence of thromboembolic events among people with COVID-19 were predominantly conducted during the early period of the pandemic, and often focused on patients hospitalised with COVID-19 only. Evidence from routinely collected health data suggested that increased risk of VTE events can be observed up to 7 to 49 weeks post-COVID-19, with estimates varying for the individual component of VTE (e.g. deep vein thrombosis (DVT), pulmonary embolism (PE)) and data sources^3–7^. Studies have also suggested that the elevated risk of thromboembolic events associated with SARS-CoV-2 infection may be attenuated following SARS-CoV-2 vaccination^8^.

SARS-CoV-2 variants have changed over time. On 26 November 2021, the European Centre for Disease Prevention and Control classified the Omicron B.1.1.529 variant as a variant of concern due to concerns regarding immune escape and potentially increased transmissibility compared to the SARS-CoV-2 delta variant^9^. It was uncertain however to what extent evidence generated by previous studies was generalisable to SARS-CoV-2 infection during the Omicron period. Relatively few studies reported rates of VTE and ATE with Omicron, particularly in the context of exposure to either prior SARS-CoV-2 infection or prior SARS-CoV-2 vaccinations^10^. We therefore focused on the Omicron-dominant period to evaluate thromboembolic event rates in the context of widespread vaccination, prior infection, and changes in clinical management.

We conducted a large multi-data source observational cohort study to estimate the incidence rates of thromboembolic events and cardiovascular events among patients with COVID-19 during Omicron, and to assess how these rates compared with the pre-pandemic general population. A pre-pandemic comparator was selected to provide a stable reference for background incidence, unaffected by SARS-CoV-2 circulation or pandemic-related changes in healthcare delivery. The conceptual idea of the current study is based on a previous project conducted at the earlier stage of the pandemic by the same research group^5,6,11,12^.

This study was conducted through the ‘Data Analysis and Real World Interrogation Network’ (DARWIN EU^®^), which is an initiative created by the European Medicines Agency (EMA) to generate timely evidence from healthcare data sources from across Europe^13^, and was one of the use- cases of the HealthData@EU pilot project with the European Health Data Space (EHDS)^14^ to test and inform HealthData@EU frameworks^14^.

## Results

### Study participants

The pre-pandemic cohort included a total of 7.6 million individuals across included data sources (CPRD GOLD: 5,278,189, IPCI: 1,589,237, SIDIAP: 746,737). We identified a total of 0.8 million people with COVID-19 during the Omicron period (refer as “COVID-19 Omicron cohort” below), with 248,847 from CPRD GOLD; 330,200 from IPCI; and 200,563 from SIDIAP. Baseline characteristics of the pre-pandemic and COVID-19 Omicron cohorts are provided for all data sources in Table 1.

**Table 1:** Baseline characteristics of the background population and COVID-19 Omicron cohort, by data source.

Variable	CPRD GOLD		IPCI		SIDIAP	
	Pre-pandemic	COVID-19 Omicron	Pre-pandemic	COVID-19 Omicron	Pre-pandemic	COVID-19 Omicron
Number of people	5,278,189	248,847	1,589,237	330,200	746,737	200,563
Number of records	5,278,189	250,463	1,589,237	339,552	746,737	212,578
Sex: Female	2,673,727 (51%)	142,704 (57%)	810,595 (51%)	185,924 (55%)	376,562 (50%)	118,509 (56%)
Age median [Q25—Q75]	38 [21–57]	37 [23–52]	39 [20–58]	37 [20–53]	40 [23–57]	43 [25–59]
Age group						
0 to 19	1,243,130 (24%)	46,814 (19%)	384,036 (24%)	80,763 (24%)	161,407 (22%)	40,466 (19%)
20 to 44	1,813,022 (34%)	111,723 (45%)	508,741 (32%)	127,347 (38%)	264,623 (35%)	72,581 (34%)
45 to 54	720,619 (14%)	38,214 (15%)	226,490 (14%)	51,360 (15%)	107,638 (14%)	35,559 (17%)
55 to 64	603,147 (11%)	29,856 (12%)	195,946 (12%)	39,273 (12%)	82,670 (11%)	23,931 (11%)
65 to 74	476,148 (9%)	13,972 (6%)	159,007 (10%)	22,965 (7%)	64,434 (9%)	17,261 (8%)
75 to 84	284,160 (5%)	6834 (3%)	84,893 (5%)	13,166 (4%)	43,216 (6%)	13,479 (6%)
85 to 150	137,960 (3%)	3050 (1%)	30,124 (2%)	4678 (1%)	22,749 (3%)	9301 (4%)
Immunocompromised: Yes	49,957 (1%)	3730 (1%)	17,058 (1%)	4967 (1%)	7009 (1%)	4249 (2%)
Vaccine doses						
0		50,641 (20%)		185,861 (55%)		31,276 (15%)
1		14,127 (6%)		33,994 (10%)		18,227 (9%)
2		64,828 (26%)		68,010 (20%)		84,017 (40%)
3		116,775 (47%)		43,820 (13%)		73,108 (34%)
≥4		4092 (2%)		7867 (2%)		5950 (3%)
History of COVID-19*		22,979 (9%)		41,206 (12%)		38,225 (18%)
Comorbidities						
Acute myocardial infarction	43,388 (1%)	2175 (1%)	12,804 (1%)	3323 (1%)	5437 (1%)	2416 (1%)
Asthma	362,427 (7%)	26,857 (11%)	55,858 (4%)	21,226 (6%)	29,742 (4%)	15,192 (7%)
Atrial fibrillation	72,407 (1%)	3164 (1%)	18,255 (1%)	5058 (1%)	15,337 (2%)	6564 (3%)
Cancer	123,236 (2%)	6351 (3%)	42,581 (3%)	12,597 (4%)	30,603 (4%)	12,998 (6%)
Chronic kidney disease	161,630 (3%)	5031 (2%)	706 (0%)	238 (0%)	24,331 (3%)	10,691 (5%)
Chronic liver disease	8129 (0%)	555 (0%)	1200 (0%)	324 (0%)	5481 (1%)	1752 (1%)
Chronic obstructive pulmonary disease	82,804 (2%)	3830 (2%)	23,359 (1%)	4682 (1%)	17,732 (2%)	6774 (3%)
Dementia	27,380 (1%)	1069 (0%)	5767 (0%)	1092 (0%)	8633 (1%)	4154 (2%)
Diabetes	190,935 (4%)	10,472 (4%)	55,408 (3%)	13,290 (4%)	46,219 (6%)	17,436 (8%)
Heart failure	33,063 (1%)	1487 (1%)	10,241 (1%)	2554 (1%)	13,279 (2%)	5819 (3%)
Hypertension	430,557 (8%)	18,608 (7%)	125,353 (8%)	28,903 (9%)	108,680 (15%)	38,901 (18%)
Ischemic stroke	9662 (0%)	413 (0%)	4433 (0%)	1098 (0%)	8135 (1%)	3532 (2%)
Obesity	894,021 (17%)	58,801 (23%)	85,117 (5%)	28,986 (9%)	139,689 (19%)	50,204 (24%)
Rheumatoid arthritis	20,926 (0%)	1342 (1%)	8288 (1%)	2637 (1%)	2628 (0%)	1167 (1%)
Medications use in 6 months before index						
Anti-inflammatory drugs	1,108,251 (21%)	64,565 (26%)	202,010 (13%)	48,792 (14%)	239,730 (32%)	100,171 (47%)
Antineoplastic drugs	235,013 (4%)	18,227 (7%)	81,081 (5%)	28,906 (9%)	20,978 (3%)	10,063 (5%)
Antithrombotics drugs	339,680 (6%)	13,873 (6%)	99,955 (6%)	22,141 (7%)	63,219 (8%)	23,848 (11%)
Glucocorticoids	652,311 (12%)	35,228 (14%)	178,871 (11%)	44,973 (13%)	80,674 (11%)	33,108 (16%)
Lipid-lowering drugs	522,394 (10%)	20,460 (8%)	129,285 (8%)	25,661 (8%)	95,667 (13%)	31,592 (15%)
Renin-angiotensin-aldosterone system inhibitors	471,628 (9%)	19,975 (8%)	125,977 (8%)	26,288 (8%)	93,870 (13%)	30,982 (15%)

Characteristics of the participants in the study cohorts used for the primary analyses. We required participants had at least one year of prior history before index date in the data source. Pre-Pandemic refers to the general population cohort 2017–2019. Age is presented as median [interquartile range-. * History of COVID-19 refers to had SARS-CoV-2 infection since the start of the COVID-19 to the 90 days prior to index date by definition.

The pre-pandemic cohort had a median age of 38 to 40 years old, and 50–51% of which were female. Among COVID-19 Omicron cohort, 55–57% were female. People in the COVID-19 Omicron cohort was slightly older in SIDIAP compared with CPRD and IPCI (median age of 37 and interquartile range (IQR) [23–52] in CPRD GOLD, 37 [20–53] in IPCI, and 43 [25–59] in SIDIAP). Approximately 1% of people were immunocompromised at index date among the pre-pandemic cohort.

In CPRD GOLD, 9% of the COVID-19 Omicron cohort had a previous SARS-CoV-2 infection (more than 90 days before index date), and 80% had received at least one dose of a COVID-19 vaccine. In IPCI, 12% of people with COVID-19 had previous SARS-CoV-2 infection, and 45% had received at least one prior dose of a COVID-19 vaccine at index date. In SIDIAP, 18% of people with COVID-19 had previous SARS-CoV-2 infection, and 85% had received at least one prior dose of a COVID-19 vaccine at index date. Among all three data sources, there were 1–2% of eligible COVID-19 cases that were classified as immunocompromised at index date.

In CPRD GOLD, 19,931 ATE and 19,009 VTE events were observed in the pre-pandemic cohorts, with 146 ATE and 238 VTE events observed within 180 days after COVID-19 during the Omicron period. In IPCI, 34,294 ATE and 9007 VTE events were observed in the pre-pandemic cohort, with 970 ATE and 391 VTE events within 180 days after infection. In SIDIAP, 7647 ATE and 2730 VTE events were observed in the pre-pandemic cohort, with 610 ATE and 295 VTE events observed within 180 days after infection.

### Main results

The overall crude incidence rates (IRs) of the study outcomes for the pre-pandemic general population cohort and the pre-pandemic immunocompromised cohort are shown in Table 2. Crude IRs in the pre-pandemic population varied by data source. For example, the crude IR per 100,000 person years (pys) for VTE was 136 [95% confidence interval 131–141] in SIDIAP, 167 [164–169] in CPRD GOLD, and 264 [259–270] in IPCI, whilst the crude IR for ATE was 175 [173–177] in CPRD GOLD, 381 [133–143] in SIDIAP, and 1,001 [991–1012] in IPCI. For all participating data sources, the IRs among those immunocompromised cohort were higher compared to the general population. For example, the crude IR of pulmonary embolism (PE) was 62 [58–65] in SIDIAP, 77 [75–78] in CPRD GOLD, 134 [130–138] in IPCI in the pre-pandemic general population, whilst the IR among the pre-pandemic immunocompromised cohort was 811 [688–949], 572 [531–615], and 1012 [923–1107] in SIDIAP, CPRD GOLD, and IPCI respectively. In the pre-pandemic cohort, we observed that the IRs of most of the study events increased with age, and rates were higher among male than female in the same age group among most data sources. (Appendix3, Figure S1)

**Table 2:** Crude incidence rates per 100,000 person-years and 95% confidence interval among the pre-pandemic cohorts of each outcome by data source.

Outcome	CPRD GOLD		IPCI		SIDIAP	
	General-population	Immunocompromised	General-population	Immunocompromised	General-population	Immunocompromised
Number of people	5,278,189	49,957	1,589,237	17,058	746,737	7,009
Acute myocardial infarction	143 [140–145]	354 [322–389]	529 [521–537]	1366 [1263–1476]	137 [132–142]	536 [437–650]
Angina (broad definition)	1527 [1520–1534]	3093 [2997–3191]	763 [754–773]	2164 [2034–2300]	1346 [1330–1362]	2432 [2216–2664]
Angina (narrow definition)	94 [92–95]	214 [190–241]	763 [754–773]	2164 [2034–2300]	138 [133–143]	484 [390–592]
Arterial thromboembolism	175 [173–177]	423 [388–460]	1001 [991–1012]	2752 [2606–2904]	381 [372–389]	1222 [1071–1389]
Cerebral venous sinus thrombosis	1 [1–2]	NA [NA-NA]	0 [0–0]	0 [0–8]	1 [0–1]	0 [0–19]
Deep vein thrombosis	96 [94–98]	317 [286–349]	137 [133–141]	486 [425–553]	91 [87–95]	543 [444–658]
Haemorrhagic stroke	17 [16–18]	34 [24–45]	20 [19–22]	27 [15–47]	55 [52–58]	182 [127–253]
Heart failure	185 [183–188]	520 [482–562]	513 [505–520]	2128 [1998–2264]	704 [692–715]	3116 [2871–3376]
Ischemic stroke	30 [29–31]	63 [50–78]	189 [184–193]	429 [372–493]	228 [222–235]	640 [532–764]
Major cardiovascular event	365 [361–368]	933 [881–988]	1579 [1566–1592]	5073 [4874–5277]	1097 [1082–1111]	4416 [4124–4723]
Portal vein thrombosis	2 [2–2]	13 [7–20]	0 [0–0]	0 [0–8]	15 [14–17]	99 [59–154]
Pulmonary embolism	77 [75–78]	272 [245–303]	134 [130–138]	554 [489–626]	62 [58–65]	338 [261–431]
Stroke	133 [131–135]	305 [275–337]	557 [550–565]	1412 [1307–1523]	267 [260–274]	754 [637–888]
Sudden cardiac death	8 [8–9]	25 [17–35]	2 [1–2]	NA [NA-NA]	1 [1–2]	NA [NA-NA]
Venous thromboembolism	167 [164–169]	572 [531–615]	264 [259–270]	1012 [923–1107]	136 [131–141]	811 [688–949]
Ventricular arrhythmia cardiac arrest	25 [24–26]	60 [48–76]	34 [32–36]	53 [34–78]	39 [36–42]	114 [72–173]

Crude incidence rates of study outcomes among the pre-pandemic general population cohort and the immunocompromised sub cohort. Angina (Narrow definition) included only angina pectoris, whiles the broad definition also included chest pain. NA suggested that the outcome number were below five and suppressed.

**Fig. 1:**
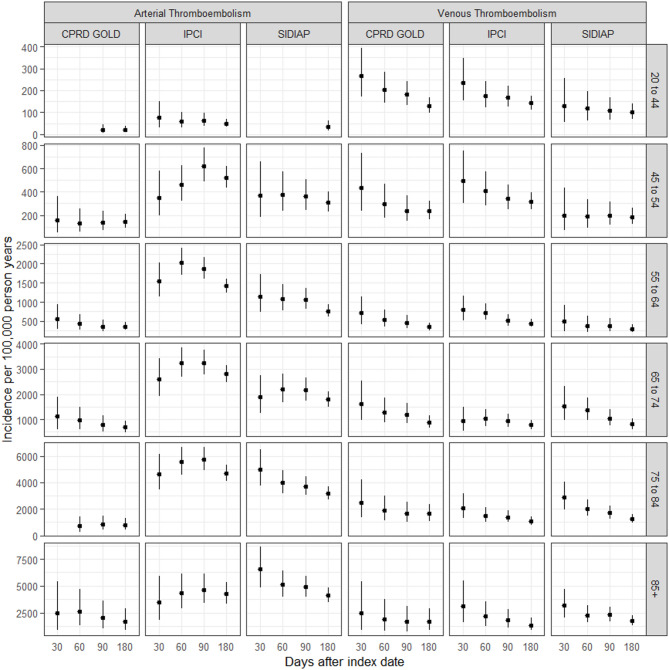
Age group specific incidence rate of VTE and ATE in the COVID-19 Omicron cohort within 30-, 60-, and 90- and 180-days. Events with fewer than 5 occurrences were omitted for privacy reasons. Point estimates with 95% confidence intervals.

The age group specific IR of VTE and ATE within 30-, 60-, and 90- and 180-days after index among the COVID-19 Omicron cohort are presented in Fig. 1. We observed that in most age groups, the IR of both VTE and ATE were higher within 30 days after COVID-19, and the estimates gradually declined during the 60-, 90-, and 180- days after infection. For example, In CPRD GOLD, the incidence rate for VTE among the 55 to 64 year old group was 717 [425–1133] per 100,000 pys within 30 days of SARS-CoV-2 infection vs. 348 [253–465] within 180 days. However, the observed IRs increased with time for ATE in IPCI. A similar pattern of changes was observed for individual conditions of VTE and ATE.

In the stratified analysis, we observed higher IR of VTE among people without prior SARS-CoV-2 infection compared to those with no infection history across all data sources, especially during the first 90 days. (Appendix 3, Figures S2) When stratified by the number of vaccine doses received, individuals who received one dose of the COVID-19 vaccine had lower IR of both VTE and ATE. However, many of the stratified estimates showed very wide confidence intervals due to the small strata size. (Appendix 3, Figures S3)

**Fig. 2:**
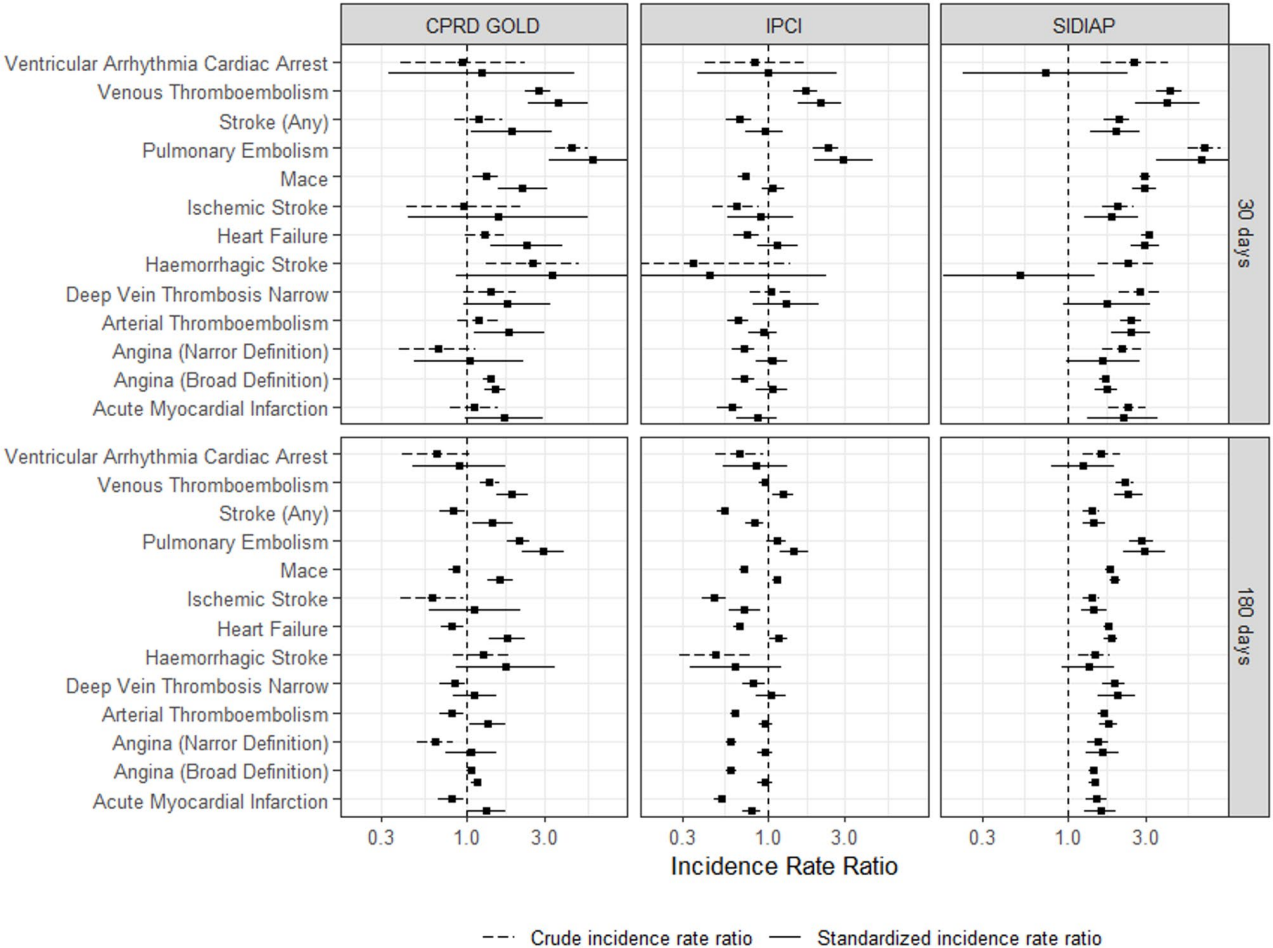
Crude and Age-sex standardised incidence ratios of study events during the 30 and 180-days after having COVID-19 in Omicron period. Crude and age-sex standardized incidence ratios with 95% confidence intervals (CIs) were estimated. Events with less than 5 occurrences have been omitted for privacy reasons.

Crude incidence rate ratios (IRRs) and age-sex standardised incidence ratios (SIRs) of study events during the 30 and 180 days after COVID-19 in Omicron period are presented in Fig. 2. In CPRD GOLD, compared with the pre-pandemic cohort, there were higher SIR of VTE, ATE, and individual events of pulmonary embolism, heart failure, and stroke during the 30 days after SARS-CoV-2 infection during the Omicron period. In IPCI, higher SIR of VTE was observed, mostly contributed by pulmonary embolism. In SIDIAP, higher SIRs were observed after SARS-CoV-2 infection for most of the study outcomes except arrhythmia and haemorrhagic stroke. The SIR was larger within 30 days of infection and persisted up to 180 days, though the estimate was slightly attenuated over time. For example, in CPRD GOLD, the SIR of VTE at 30 days after index was 3.61 [2.45–5.53] and dropped to 1.88 [1.52–2.34]. In SIDIAP, the SIR at 30- and 180- days after index was 4.10 [2.62–6.41] and 2.37 [1.92–2.92], respectively.

**Fig. 3:**
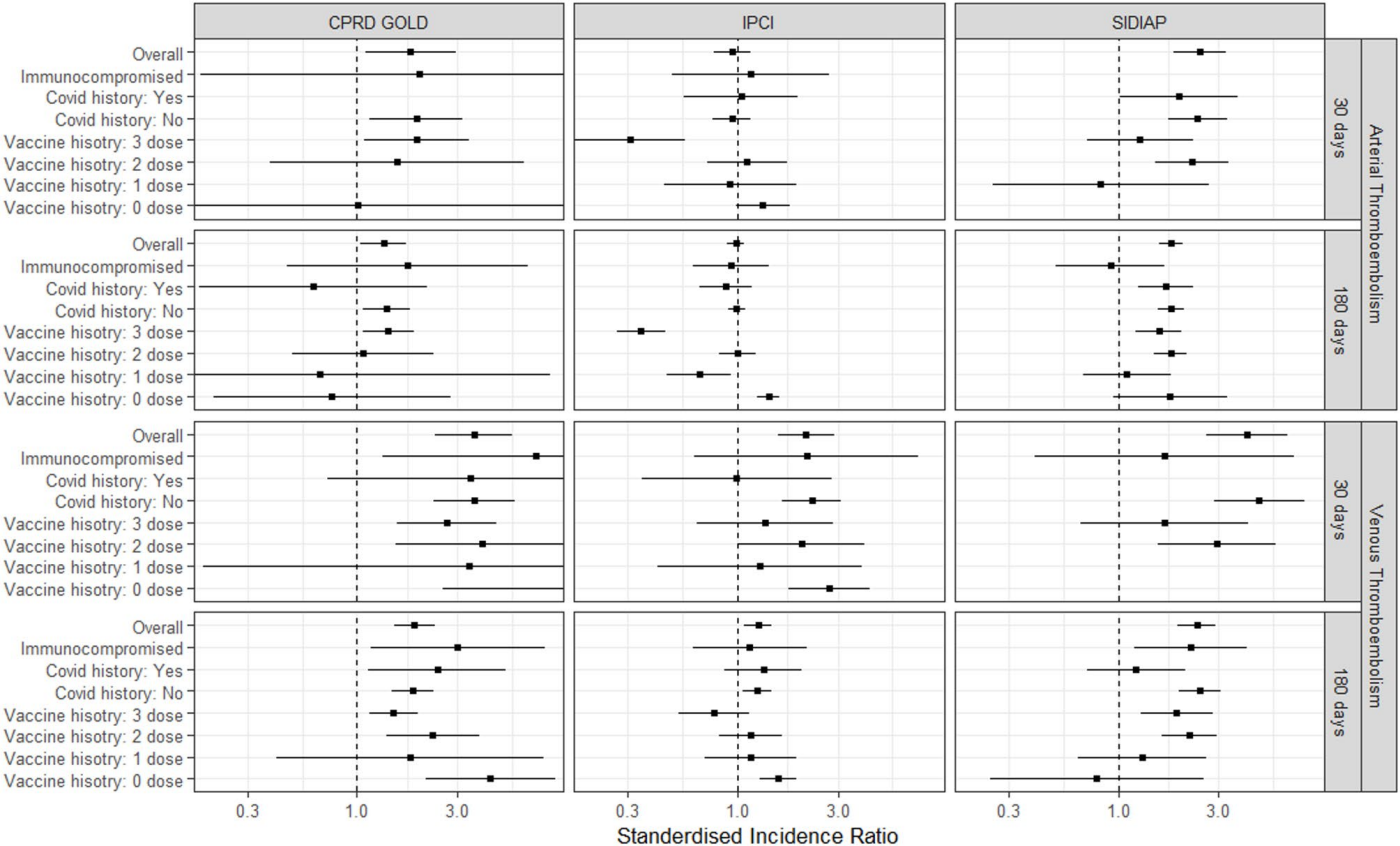
Standardised incidence rate ratio of ATE and VTE during the 30- and 180-days after infection for the COVID-19 Omicron cohort as compared to the pre-pandemic cohort, stratified by vaccine and covid status, and immunocompromised status. Immunocompromised results were compared to the pre-pandemic immunocompromised cohort. Empty rows suggested that the outcome number were below five and been suppressed.

Standardised incidence rate ratios were estimated for stratifications of COVID-19 history, vaccine status, and immunocompromised status (Fig. 3). Among individuals who were immunocompromised at the index date, SIR for VTE were also higher in CPRD GOLD up to 180 days post index, and in SIDIAP at 180 days post index. When stratified by prior SARS-CoV-2 infection, the SIR for VTE was higher among those without a history of infection and persisted up to 180 days post-index, across all data sources. However, the precision of these estimates was limited in several strata due to small sample sizes.

### Sensitivity analysis

When restricted to first-ever events, we observed that SIR estimates moved away from the null in all included data sources, the magnitude of which differed by outcome and data source. (Appendix3, figure S6)

## Discussion

We estimated the incidence rates of VTE, ATE, and cardiovascular events among people with COVID-19 during the Omicron period compared to the pre-pandemic population. While the incidence rates of certain thromboembolic events were numerically lower than during the first 30 days after COVID-19, they remained significantly higher than those in the general population up to six months post-infection. Higher SIRs were also observed for individual conditions included in the composite outcomes, including pulmonary embolism and heart failure.

An increased risk of VTE during SARS-CoV-2 infection has been reported in several studies sin the start of the pandemic^12,15,16^. The mechanisms between COVID-19 and thrombosis involves increased cytokines and activation of platelets, dysfunction of endothelial cell, and other complex interactions between the innate immune response, the coagulation and fibrinolytic pathways, and the vascular endothelium^17^.

In late 2021, the study “Natural history of coagulopathy and use of anti-thrombotic agents in COVID-19 patients and persons vaccinated against SARS-CoV-2” was conducted (EUPAS40414) by the same research group^5,6,11,12,18^, using data from 5 European countries (the Netherlands, Italy, France, Germany, Spain, and the UK). In the main analysis of the previous study, a similar pre-pandemic population during year 2017–2019 was constructed without the requirement of 1 year visibility in the data source before index. The incidence rates of the pre-pandemic population estimated from both studies were overall similar, with some differences in IPCI. For example, the IR of ATE from the main analysis of current study was 1,001 [991–1012] per 100,000 person-years, 628 [619–636] in the sensitivity analysis, and 319 [313–326] in the previous study in IPCI. In CPRD GOLD, the IRs were 175 [173–177] and 172 [170–175] from current and previous study, respectively. This discordance in background rates resulted from the changes of phenotyping of study outcomes, changes in the source codes which lead to changes in mapping of standardised vocabulary, differences in the way that the analytical codes were programmed, and a combination of all the above.

Cardiovascular complications have been described after infection during the first and second waves of COVID-19. While most studies examined the risk during the acute phase after infection (within 30 days)^19,20^, some studies followed people with COVID-19 for a longer-term, from 90 days after infection^5^, to 180 days or 12-months^4,21,22^. In the current study, we found that the incidence rates of some of the included thromboembolic events, while numerically lower than the acute phase, were still significantly higher than the pre-pandemic population up to 6-months during the Omicron period. In a self-controlled case series study using national registries from Sweden prior to the Omicron variant, the risk of deep vein thrombosis and pulmonary embolism was estimated during the 180 days after COVID-19. The study found an increased risk of deep vein thrombosis up to 90 days after SARS-CoV-2 infection and of pulmonary embolism up to 180 days after^4^. The incidence rate ratio decreased after the first 30 days for both outcomes. Another study, using electronic health records up to December 2020 from the UK, showed the highest risk of VTE during the first week after infection. While the risk decreased over time, they still observed an increased hazard ratio of VTE of 1.80 (95% CI 1.50–2.17) up to weeks 27 to 49 after infection^23^.

In our study, the stratified analysis of SIR showed higher rates of VTE, PE, heart failure, ATE (CPRD GOLD) among people without previous SARS-CoV-2 infection history, but not among those with prior SARS-CoV-2 infection. However, the number of people with prior SARS-CoV-2 infection was much smaller, thus the 95% confidence interval of the rate ratio was large and imprecise. Previous studies among healthcare worker found that previous infection can provide addition protection against Omicron on top of vaccination^24,25^. The immunity induced from previous infection could also confer protection against thrombosis outcomes.

We observed higher SIR of VTE up to 180 days after COVID-19 during the Omicron period in the CPRD GOLD and SIDIAP data sources, which was in line with the overall unstratified analysis. Event number of ATE was very low which led to wide and imprecise confidence intervals. In a matched cohort study conducted using health administrative data from Canada, the authors found that the risk of VTE was not statistically significantly different for people with immune-mediated inflammatory diseases as compared to people without immune-mediated inflammatory diseases^26^.

The current study utilised data sources from multiple European countries and was conducted with federated analyses manner. We focused on the period when Omicron variant was dominant, and able to examine the incidence rates of outcomes up to 6 months post-infection in various stratification of population.

The findings of this study need to be interpreted in the context of several limitations. First results may not be generalised beyond the data sources included. During the pandemic, the national and regional regulations, testing recommendations, and clinical practice of testing changed over time. For example, in the UK, from January 2022 onwards, individuals testing positive with a lateral flow test were no longer required to confirm the result with a PCR test. Although reporting to the UK National Health Service was recommended, it remains unclear what proportion of these home test results were reported. Even when reported, the potential bias from self-reported data cannot be neglected. Additionally, different vaccines were approved and rolled out with various speed and priority groups as per country-specific schedules. Therefore, findings reported from this study may also not be generalisable beyond the specific study period.

The study used the pre-pandemic comparator cohort, which does not fully account for secular trends, including changes in healthcare utilisation, diagnostic intensity, and population risk profiles that occurred during the pandemic period. Consequently, observed differences may partly reflect temporal trends rather than associations with SARS-CoV-2 infection. However, defining a truly unexposed population during the Omicron period is challenging due to widespread, and often undocumented infections.

The analyses calculated crude incidence rates and standardised incidence rates accounting for age and sex only. Although the characteristics of each cohort and stratification group was inspected, in the analyses we did not account for other possible confounders including socioeconomic status, presence of comorbidities or use of certain medicines. Therefore, the observed rate ratios should be interpreted with caution as measures of association rather than causal effects. The multiple analyses performed and stratification by age and sex can lead to spurious findings. In addition, the immunocompromised status was defined at baseline and not updated over time, which may have resulted in misclassification, particularly in the pre-pandemic cohort with longer follow-up. We did not have access to variant-level data at the individual level. Therefore, some misclassification of variant exposure during the transition period cannot be excluded.

We observed heterogeneity in background incidence rates across the participating data sources. These differences likely reflect variation in underlying population demographics, coding practices, and healthcare systems. To account for this, we estimated incidence rates and standardised incidence ratios within each data source separately, thereby enabling comparisons relative to internal baseline rates. As such, comparisons of absolute incidence across data sources should be interpreted with caution, and emphasis should be placed on within-data source estimates. Differences in data capture may also contribute to the observed heterogeneity. For example, inpatient data were not available in the CPRD GOLD data. If individuals developed severe outcomes which required emergency department or hospital visit, we might not be able to capture these events, leading to an underestimate of incidence rate. However, previous studies found that cases of VTE identified form CPRD data had high validity^27,28^, and the impact on incidence rate ratio were minimal as we expected similar level of misclassification for the general population and the COVID-19 cohort.

As one of the use cases of the Healthdata@EU pilot project, this study demonstrated the advantages and challenges of using routinely collected health data mapped to the OMOP common data model to conducted cross-boarder federated analyses within Europe.

## Methods

### Study design and data sources

We conducted a population-based cohort study using routinely collected, de-identified electronic health records from three European countries: the UK (Clinical Practice Research Datalink, CPRD GOLD)^29^, the Netherlands (Integrated Primary Care Information Project, IPCI)^30^, and Spain (Sistema d’Informació per al Desenvolupament de la Investigació en Atenció Primària, SIDIAP)^31^.

CPRD GOLD is a population-level primary care data source that comprised 3.1 million active participants predominantly covering practices in Scotland and Wales^32^. However, in CPRD GOLD, the contributing practice base has changed substantially over time, leading to a decrease in the total number of active patients in the data source^29^. The number of active is estimated to have decreased by 32% between 2017 and end of 2022.

The IPCI data source contains electronic healthcare records collected from patients registered with general practices in the Netherlands^33^. SIDIAP represents around 80% of the population living in Catalonia, an autonomous community in northeastern Spain, and includes primary care records with linked hospitalisation and intensive care use information^31^. The sizes of the IPCI and SIDIAP data sources remained relatively stable over the same period.

All three data sources include information on patient demographics, comorbidities, medicine use, laboratory measurements, clinical measurements, and were linked to national or regional registry data on COVID-19 vaccination. All data sources were mapped to the Observational Medical Outcomes Partnership (OMOP) common data model (CDM) to enable federated analytics without sharing patient level data^34^. We used a 1,000,000 random sample of people in SIDIAP due to limited computing resources. All analyses were conducted separately within each data source to account for differences in population characteristics, data capture, and healthcare systems, and results were not pooled across data sources.

### Study populations

The study population included a “pre-pandemic” cohort and a ‘COVID-19 Omicron’ cohort. The pre-pandemic cohort included people from the general population of each data source during 2017 to 2019. The index date of each individual was 1st January 2017, or the date when the individual had 365 days of data availability in the data source. We used the pre-pandemic population instead of a concurrent SARS-CoV-2-negative comparator due to the high prevalence of asymptomatic or unrecorded infections and potential misclassification of infection status. In addition, differences in healthcare-seeking behaviour and testing practices during the pandemic may introduce selection bias in identifying truly uninfected individuals.

The ‘COVID-19 Omicron’ cohort included people with COVID-19 when Omicron was dominant, defined by having either a positive test result for SARS-CoV-2 or a clinical coded diagnosis of COVID-19 on or after 1st December 2021, and had no positive test result for SARS-CoV-2 or clinical diagnosis of COVID-19 in the prior 3 months. The Omicron-dominant period was defined as based on European genomic surveillance data indicating that the Omicron (B.1.1.529) variant became the predominant circulating strain during this time in the UK, the Netherlands, and Spain^35,36^. Index date was the date of diagnosis or test. We required individuals to have at least 365 days of data availability prior to index date.

In both cohorts, people were followed-up from the index date until the earliest of the following: 31 December 2019 for the pre-pandemic cohort; 180 days after index date of the COVID-19 cohort; end of their observation—i.e., date of data extraction (December 15, 2022 for CPRD GOLD; June 30, 2023 for IPCI; June 30, 2023 for SIDIAP); death, leaving the general practitioner (GP) practice or the practice stopping contribution of data to data source (for CPRD GOLD).

Among both the pre-pandemic cohort and the COVID-19 Omicron cohort, we identified a subpopulation of people who were immunocompromised at index date. This status was not updated during follow-up. The clinical codes used to define COVID-19 and definition of immunocompromised are available in the appendix.

### Outcomes

We included VTE, ATE, and cardiovascular events as study outcomes. VTE were identified by diagnostic codes for pulmonary embolism or deep vein thrombosis. ATE were identified by an acute myocardial infarction or acute ischemic stroke. We also included cardiovascular events of heart failure, cardiac arrhythmia, angina, and major cardiovascular events (MACE). MACE was defined by heart failure, or acute myocardial infarction, or stroke, or the occurrence of sudden cardiac death. Clinical code lists for the definition of study outcomes are available in the appendix.

### Statistical analysis

We reported the observed characteristics of each cohort within participated data sources. For the COVID-19 Omicron cohort, we assessed the number of COVID-19 vaccines each person received prior to the index date. We required at least 21 days between two consecutive vaccination records. Demographic characteristics, health conditions and medication use before the cohort index date were reported.

We calculated incidence rates with 95% confidence intervals of study outcomes among the pre-pandemic cohort. Among the COVID-19 Omicron cohort, incidence rates during the 30-, 60-, and 90- and 180-days following the index date were estimated. Age group and sex stratified incidence rates were reported.

We estimated the crude incidence rate ratios (IRRs) for each event with 95% confidence intervals for the COVID-19 cohorts compared against the pre-pandemic cohort. To calculate standardised incidence rate ratios (SIRs) and 95% confidence intervals, we used indirect standardisation by age groups and sex to the pre-pandemic cohort as the standard population. A standardised incidence rate ratio above 1 indicates that the observed rate for a specific outcome is higher than what is expected in the pre-pandemic population. Analyses were stratified by prior COVID-19 diagnosis, and prior SARS-CoV-2 vaccination status. For patients who were immunocompromised on their index date, the IRR and SIR were calculated against the incidence rates among the pre-pandemic immunocompromised cohort.

In the main analysis, we applied a washout period of 180 days to define incident event, we restricted to the first-ever event in the sensitivity analysis. All analyses were conducted in R (4.3.2) using the IncidencePrevalence^37^ and CohortCharacteristics^38^ R packages.

The study was registered with HMA-EMA Catalogue of RWD studies (EU PAS number EUPAS106679, https://catalogues.ema.europa.eu/node/3837/). All our analytical code is available for review in a dedicated Github repository at: https://github.com/darwin-eu-studies/P2-C3-001-EhdsCoagulopathy/blob/main/Study/mainStudy.

## Supplementary Information

Below is the link to the electronic supplementary material.

Supplementary Material 1.

## Data Availability

The data that support the findings of this study are available from the Clinical Practice Research Datalink (CPRD), the Clinical Research Ethics committee of Fundació Institut Universitari per a la recerca a l’Atenció Primària de Salut Jordi Gol i Gurina (IDIAPJGol), and the Integrated Primary Care Information (IPCI) but restrictions apply to the availability of these data, which were used under license for the current study, and so are not publicly available. Data are however available from the corresponding author upon reasonable request and with permission of CPRD, IDIAPJGol and IPCI.
