# Supplementary material for: Venous and arterial thromboembolic events after COVID-19 during the Omicron period in three European countries
Source: Sci Rep. 2026 May 15;16:22154. doi: 10.1038/s41598-026-51445-7 (PMC13369811; doi:10.1038/s41598-026-51445-7)

Contents

[Appendix 1. Code list of COVID-19 definition 1](#_Toc186991993)

[Appendix 2. Code list of study outcomes 5](#_Toc186991994)

[Appendix 3. Definition of immunocompromised status at the index date 12](#_Toc186991995)

[Appendix 3. Supplement tables and figures 13](#_Toc186991996)

### Appendix 1. Code list of COVID-19 definition

| **Concept Id** | **Concept name** |  | domain |
| --- | --- | --- | --- |
| 3661405 | Acute bronchitis caused by SARS-CoV-2 | COVID Diagnosis | Condition |
| 3655976 | Acute hypoxemic respiratory failure due to disease caused by Severe acute respiratory syndrome coronavirus 2 | COVID Diagnosis | Condition |
| 3661748 | Acute kidney injury due to disease caused by Severe acute respiratory syndrome coronavirus 2 | COVID Diagnosis | Condition |
| 3661406 | Acute respiratory distress syndrome due to disease caused by Severe acute respiratory syndrome coronavirus 2 | COVID Diagnosis | Condition |
| 3662381 | Asymptomatic SARS-CoV-2 | COVID Diagnosis | Condition |
| 756031 | Bronchitis caused by COVID-19 | COVID Diagnosis | Condition |
| 3656667 | Cardiomyopathy due to disease caused by Severe acute respiratory syndrome coronavirus 2 | COVID Diagnosis | Condition |
| 3656668 | Conjunctivitis due to disease caused by Severe acute respiratory syndrome coronavirus 2 | COVID Diagnosis | Condition |
| 439676 | Coronavirus infection | COVID Diagnosis | Condition |
| 37311061 | COVID-19 | COVID Diagnosis | Condition |
| 4100065 | Disease due to Coronaviridae | COVID Diagnosis | Condition |
| 3656669 | Dyspnea caused by Severe acute respiratory syndrome coronavirus 2 | COVID Diagnosis | Condition |
| 37310284 | Encephalopathy due to disease caused by Severe acute respiratory syndrome coronavirus 2 | COVID Diagnosis | Condition |
| 3661885 | Fever caused by Severe acute respiratory syndrome coronavirus 2 | COVID Diagnosis | Condition |
| 37310283 | Gastroenteritis caused by SARS-CoV-2 (severe acute respiratory syndrome coronavirus 2) | COVID Diagnosis | Condition |
| 37310286 | Infection of upper respiratory tract caused by Severe acute respiratory syndrome coronavirus 2 | COVID Diagnosis | Condition |
| 3663281 | Lower respiratory infection caused by SARS-CoV-2 | COVID Diagnosis | Condition |
| 3661631 | Lymphocytopenia due to Severe acute respiratory syndrome coronavirus 2 | COVID Diagnosis | Condition |
| 37310287 | Myocarditis due to disease caused by Severe acute respiratory syndrome coronavirus 2 | COVID Diagnosis | Condition |
| 37310254 | Otitis media due to disease caused by Severe acute respiratory syndrome coronavirus 2 | COVID Diagnosis | Condition |
| 704995 | Patient meets COVID-19 clinical diagnostic criteria | COVID Diagnosis | Observation |
| 700297 | Patient meets COVID-19 laboratory confirmation criterion (detection of specific RNA in a clinical specimen using a molecular amplification detection test) | COVID Diagnosis | Observation |
| 704996 | Patient meets COVID-19 laboratory diagnostic criteria | COVID Diagnosis | Observation |
| 700296 | Patient meets COVID-19 presumptive laboratory evidence criteria (detection of specific antigen in a clinical specimen, OR detection of specific antibody in serum, plasma, or whole blood indicative of a new or recent infection) | COVID Diagnosis | Observation |
| 37016927 | Pneumonia caused by Human coronavirus | COVID Diagnosis | Condition |
| 3661408 | Pneumonia caused by SARS-CoV-2 | COVID Diagnosis | Condition |
| 40479642 | Pneumonia due to Severe acute respiratory syndrome coronavirus | COVID Diagnosis | Condition |
| 756039 | Respiratory infection caused by COVID-19 | COVID Diagnosis | Condition |
| 3655977 | Rhabdomyolysis due to disease caused by Severe acute respiratory syndrome coronavirus 2 | COVID Diagnosis | Condition |
| 3655975 | Sepsis due to disease caused by Severe acute respiratory syndrome coronavirus 2 | COVID Diagnosis | Condition |
| 320651 | Severe acute respiratory syndrome | COVID Diagnosis | Condition |
| 37396171 | Severe acute respiratory syndrome of upper respiratory tract | COVID Diagnosis | Condition |
| 37311060 | Suspected COVID-19 | COVID Diagnosis | Observation |
| 3661632 | Thrombocytopenia due to Severe acute respiratory syndrome coronavirus 2 | COVID Diagnosis | Condition |
| 45763724 | Suspected coronavirus infection | COVID Diagnosis | Observation |
| 40218804 | 2019-ncov coronavirus, sars-cov-2/2019-ncov (covid-19), any technique, multiple types or subtypes (includes all targets), non-cdc | COVID19 positive test | Measurement |
| 40218805 | Cdc 2019 novel coronavirus (2019-ncov) real-time rt-pcr diagnostic panel | COVID19 positive test | Measurement |
| 44789510 | Coronavirus nucleic acid detection | COVID19 positive test | Measurement |
| 44811805 | Coronavirus nucleic acid detection assay | COVID19 positive test | Measurement |
| 45770687 | Coronavirus RNA (ribonucleic acid) detection assay | COVID19 positive test | Measurement |
| 44807536 | Coronavirus RNA (ribonucleic acid) measurement by NAAT (nucleic acid amplification test) | COVID19 positive test | Measurement |
| 3667069 | Detection of ribonucleic acid of Severe acute respiratory syndrome coronavirus 2 using polymerase chain reaction | COVID19 positive test | Observation |
| 36660491 | Human coronavirus 229E RNA [Presence] in Lower respiratory specimen by NAA with non-probe detection | COVID19 positive test | Measurement |
| 36659667 | Human coronavirus HKU1 RNA [Presence] in Lower respiratory specimen by NAA with non-probe detection | COVID19 positive test | Measurement |
| 36660329 | Human coronavirus NL63 RNA [Presence] in Lower respiratory specimen by NAA with non-probe detection | COVID19 positive test | Measurement |
| 36660364 | Human coronavirus OC43 RNA [Presence] in Lower respiratory specimen by NAA with non-probe detection | COVID19 positive test | Measurement |
| 742224 | Infectious agent antigen detection by immunoassay technique, qualitative or semiquantitative, multiple-step method; severe acute respiratory syndrome coronavirus (eg, SARS-CoV, SARS-CoV-2 [COVID-19]) (Coronavirus disease [COVID-19]) | COVID19 positive test | Measurement |
| 700360 | Infectious agent detection by nucleic acid (DNA or RNA); severe acute respiratory syndrome coronavirus 2 (SARS-CoV-2) (Coronavirus disease [COVID-19]), amplified probe technique | COVID19 positive test | Measurement |
| 742218 | Infectious disease (bacterial or viral respiratory tract infection), pathogen-specific nucleic acid (DNA or RNA), 22 targets including severe acute respiratory syndrome coronavirus 2 (SARS-CoV-2), qualitative RT-PCR, nasopharyngeal swab | COVID19 positive test | Measurement |
| 742219 | Infectious disease (bacterial or viral respiratory tract infection), pathogen-specific nucleic acid (DNA or RNA), 22 targets including severe acute respiratory syndrome coronavirus 2 (SARS-CoV-2), qualitative RT-PCR, nasopharyngeal swab | COVID19 positive test | Measurement |
| 36661384 | Influenza virus A and B and SARS-CoV-2 (COVID-19) and SARS-related CoV RNA panel - Respiratory specimen by NAA with probe detection | COVID19 positive test | Measurement |
| 36661375 | Influenza virus A and B and SARS-CoV-2 (COVID-19) identified in Respiratory specimen by NAA with probe detection | COVID19 positive test | Measurement |
| 36661376 | Influenza virus A and B RNA and SARS-CoV-2 (COVID-19) N gene panel - Respiratory specimen by NAA with probe detection | COVID19 positive test | Measurement |
| 705104 | Measurement of Coronavirus (Coronavirinae subfamily species) | COVID19 positive test | Measurement |
| 705105 | Measurement of Coronavirus (Coronavirinae subfamily species) antigen | COVID19 positive test | Measurement |
| 37310257 | Measurement of Severe acute respiratory syndrome coronavirus 2 antigen | COVID19 positive test | Measurement |
| 756055 | Measurement of Severe acute respiratory syndrome coronavirus 2 (SARS-CoV-2) | COVID19 positive test | Measurement |
| 586310 | Measurement of Severe acute respiratory syndrome coronavirus 2 (SARS-CoV-2) Genetic material using Molecular method | COVID19 positive test | Measurement |
| 704991 | Measurement of Severe acute respiratory syndrome coronavirus 2 (SARS-CoV-2) in Blood | COVID19 positive test | Measurement |
| 756029 | Measurement of Severe acute respiratory syndrome coronavirus 2 (SARS-CoV-2) in Respiratory specimen | COVID19 positive test | Measurement |
| 586307 | Measurement of Severe acute respiratory syndrome coronavirus 2 (SARS-CoV-2) in Saliva | COVID19 positive test | Measurement |
| 705107 | Measurement of Severe acute respiratory syndrome coronavirus 2 (SARS-CoV-2) in Sample from nose | COVID19 positive test | Measurement |
| 704976 | Measurement of Severe acute respiratory syndrome coronavirus 2 (SARS-CoV-2) in Sample from oropharynx | COVID19 positive test | Measurement |
| 586309 | Measurement of Severe acute respiratory syndrome coronavirus 2 (SARS-CoV-2) in Specified specimen | COVID19 positive test | Measurement |
| 756065 | Measurement of Severe acute respiratory syndrome coronavirus 2 (SARS-CoV-2) in Unspecified specimen | COVID19 positive test | Measurement |
| 702834 | Measurement of Severe acute respiratory syndrome coronavirus 2 (SARS-CoV-2) specific cell-mediated immune response in Blood | COVID19 positive test | Measurement |
| 704992 | Measurement of Severe acute respiratory syndrome coronavirus 2 (SARS-CoV-2) using Culture method | COVID19 positive test | Measurement |
| 705001 | Measurement of Severe acute respiratory syndrome coronavirus 2 (SARS-CoV-2) using Nucleic acid amplification technique | COVID19 positive test | Measurement |
| 705000 | Measurement of Severe acute respiratory syndrome coronavirus 2 (SARS-CoV-2) using Nucleic acid amplification technique in Blood | COVID19 positive test | Measurement |
| 756085 | Measurement of Severe acute respiratory syndrome coronavirus 2 (SARS-CoV-2) using Nucleic acid amplification technique in Respiratory specimen | COVID19 positive test | Measurement |
| 586308 | Measurement of Severe acute respiratory syndrome coronavirus 2 (SARS-CoV-2) using Nucleic acid amplification technique in Saliva | COVID19 positive test | Measurement |
| 705106 | Measurement of Severe acute respiratory syndrome coronavirus 2 (SARS-CoV-2) using Nucleic acid amplification technique in Sample from nose | COVID19 positive test | Measurement |
| 704975 | Measurement of Severe acute respiratory syndrome coronavirus 2 (SARS-CoV-2) using Nucleic acid amplification technique in Sample from oropharynx | COVID19 positive test | Measurement |
| 756084 | Measurement of Severe acute respiratory syndrome coronavirus 2 (SARS-CoV-2) using Nucleic acid amplification technique in Unspecified specimen | COVID19 positive test | Measurement |
| 704993 | Measurement of Severe acute respiratory syndrome coronavirus 2 (SARS-CoV-2) using Sequencing | COVID19 positive test | Measurement |
| 723477 | SARS-CoV-2 (COVID-19) Ag [Presence] in Respiratory specimen by Rapid immunoassay | COVID19 positive test | Measurement |
| 706167 | SARS-CoV-2 (COVID-19) N gene [Cycle Threshold #] in Unspecified specimen by NAA with probe detection | COVID19 positive test | Measurement |
| 706157 | SARS-CoV-2 (COVID-19) N gene [Cycle Threshold #] in Unspecified specimen by Nucleic acid amplification using CDC primer-probe set N1 | COVID19 positive test | Measurement |
| 706155 | SARS-CoV-2 (COVID-19) N gene [Cycle Threshold #] in Unspecified specimen by Nucleic acid amplification using CDC primer-probe set N2 | COVID19 positive test | Measurement |
| 715272 | SARS-CoV-2 (COVID-19) N gene [Presence] in Nasopharynx by NAA with probe detection | COVID19 positive test | Measurement |
| 757678 | SARS-CoV-2 (COVID-19) N gene [Presence] in Nose by NAA with probe detection | COVID19 positive test | Measurement |
| 706161 | SARS-CoV-2 (COVID-19) N gene [Presence] in Respiratory specimen by NAA with probe detection | COVID19 positive test | Measurement |
| 586524 | SARS-CoV-2 (COVID-19) N gene [Presence] in Respiratory specimen by Nucleic acid amplification using CDC primer-probe set N1 | COVID19 positive test | Measurement |
| 586525 | SARS-CoV-2 (COVID-19) N gene [Presence] in Respiratory specimen by Nucleic acid amplification using CDC primer-probe set N2 | COVID19 positive test | Measurement |
| 36661378 | SARS-CoV-2 (COVID-19) N gene [Presence] in Saliva (oral fluid) by NAA with probe detection | COVID19 positive test | Measurement |
| 586520 | SARS-CoV-2 (COVID-19) N gene [Presence] in Serum or Plasma by NAA with probe detection | COVID19 positive test | Measurement |
| 706175 | SARS-CoV-2 (COVID-19) N gene [Presence] in Unspecified specimen by NAA with probe detection | COVID19 positive test | Measurement |
| 706156 | SARS-CoV-2 (COVID-19) N gene [Presence] in Unspecified specimen by Nucleic acid amplification using CDC primer-probe set N1 | COVID19 positive test | Measurement |
| 706154 | SARS-CoV-2 (COVID-19) N gene [Presence] in Unspecified specimen by Nucleic acid amplification using CDC primer-probe set N2 | COVID19 positive test | Measurement |
| 723469 | SARS-CoV-2 (COVID-19) ORF1ab region [Cycle Threshold #] in Respiratory specimen by NAA with probe detection | COVID19 positive test | Measurement |
| 706168 | SARS-CoV-2 (COVID-19) ORF1ab region [Cycle Threshold #] in Unspecified specimen by NAA with probe detection | COVID19 positive test | Measurement |
| 723478 | SARS-CoV-2 (COVID-19) ORF1ab region [Presence] in Respiratory specimen by NAA with probe detection | COVID19 positive test | Measurement |
| 723464 | SARS-CoV-2 (COVID-19) ORF1ab region [Presence] in Unspecified specimen by NAA with probe detection | COVID19 positive test | Measurement |
| 586516 | SARS-CoV-2 (COVID-19) [Presence] in Unspecified specimen by Organism specific culture | COVID19 positive test | Measurement |
| 723471 | SARS-CoV-2 (COVID-19) RdRp gene [Cycle Threshold #] in Respiratory specimen by NAA with probe detection | COVID19 positive test | Measurement |
| 723470 | SARS-CoV-2 (COVID-19) RdRp gene [Cycle Threshold #] in Unspecified specimen by NAA with probe detection | COVID19 positive test | Measurement |
| 706160 | SARS-CoV-2 (COVID-19) RdRp gene [Presence] in Respiratory specimen by NAA with probe detection | COVID19 positive test | Measurement |
| 706173 | SARS-CoV-2 (COVID-19) RdRp gene [Presence] in Unspecified specimen by NAA with probe detection | COVID19 positive test | Measurement |
| 586528 | SARS-CoV-2 (COVID-19) RNA [Cycle Threshold #] in Respiratory specimen by NAA with probe detection | COVID19 positive test | Measurement |
| 586529 | SARS-CoV-2 (COVID-19) RNA [Cycle Threshold #] in Unspecified specimen by NAA with probe detection | COVID19 positive test | Measurement |
| 715262 | SARS-CoV-2 (COVID-19) RNA [Log #/volume] (viral load) in Unspecified specimen by NAA with probe detection | COVID19 positive test | Measurement |
| 706158 | SARS-CoV-2 (COVID-19) RNA panel - Respiratory specimen by NAA with probe detection | COVID19 positive test | Measurement |
| 706169 | SARS-CoV-2 (COVID-19) RNA panel - Unspecified specimen by NAA with probe detection | COVID19 positive test | Measurement |
| 723476 | SARS-CoV-2 (COVID-19) RNA [Presence] in Nasopharynx by NAA with non-probe detection | COVID19 positive test | Measurement |
| 586526 | SARS-CoV-2 (COVID-19) RNA [Presence] in Nasopharynx by NAA with probe detection | COVID19 positive test | Measurement |
| 757677 | SARS-CoV-2 (COVID-19) RNA [Presence] in Nose by NAA with probe detection | COVID19 positive test | Measurement |
| 706163 | SARS-CoV-2 (COVID-19) RNA [Presence] in Respiratory specimen by NAA with probe detection | COVID19 positive test | Measurement |
| 36661377 | SARS-CoV-2 (COVID-19) RNA [Presence] in Respiratory specimen by Sequencing | COVID19 positive test | Measurement |
| 715260 | SARS-CoV-2 (COVID-19) RNA [Presence] in Saliva (oral fluid) by NAA with probe detection | COVID19 positive test | Measurement |
| 715261 | SARS-CoV-2 (COVID-19) RNA [Presence] in Saliva (oral fluid) by Sequencing | COVID19 positive test | Measurement |
| 723463 | SARS-CoV-2 (COVID-19) RNA [Presence] in Serum or Plasma by NAA with probe detection | COVID19 positive test | Measurement |
| 706170 | SARS-CoV-2 (COVID-19) RNA [Presence] in Unspecified specimen by NAA with probe detection | COVID19 positive test | Measurement |
| 723467 | SARS-CoV-2 (COVID-19) S gene [Cycle Threshold #] in Respiratory specimen by NAA with probe detection | COVID19 positive test | Measurement |
| 723468 | SARS-CoV-2 (COVID-19) S gene [Cycle Threshold #] in Unspecified specimen by NAA with probe detection | COVID19 positive test | Measurement |
| 723465 | SARS-CoV-2 (COVID-19) S gene [Presence] in Respiratory specimen by NAA with probe detection | COVID19 positive test | Measurement |
| 586519 | SARS-CoV-2 (COVID-19) S gene [Presence] in Serum or Plasma by NAA with probe detection | COVID19 positive test | Measurement |
| 723466 | SARS-CoV-2 (COVID-19) S gene [Presence] in Unspecified specimen by NAA with probe detection | COVID19 positive test | Measurement |
| 586517 | SARS-CoV-2 (COVID-19) whole genome [Nucleotide sequence] in Isolate by Sequencing | COVID19 positive test | Measurement |
| 757685 | SARS-CoV+SARS-CoV-2 (COVID-19) Ag [Presence] in Respiratory specimen by Rapid immunoassay | COVID19 positive test | Measurement |
| 706172 | SARS-like coronavirus N gene [Cycle Threshold #] in Unspecified specimen by NAA with probe detection | COVID19 positive test | Measurement |
| 706171 | SARS-like coronavirus N gene [Presence] in Unspecified specimen by NAA with probe detection | COVID19 positive test | Measurement |
| 706166 | SARS-related coronavirus E gene [Cycle Threshold #] in Unspecified specimen by NAA with probe detection | COVID19 positive test | Measurement |
| 586523 | SARS-related coronavirus E gene [Presence] in Respiratory specimen by NAA with probe detection | COVID19 positive test | Measurement |
| 586518 | SARS-related coronavirus E gene [Presence] in Serum or Plasma by NAA with probe detection | COVID19 positive test | Measurement |
| 706174 | SARS-related coronavirus E gene [Presence] in Unspecified specimen by NAA with probe detection | COVID19 positive test | Measurement |
| 706159 | SARS-related coronavirus+MERS coronavirus RNA [Presence] in Respiratory specimen by NAA with probe detection | COVID19 positive test | Measurement |
| 706165 | SARS-related coronavirus RNA [Presence] in Respiratory specimen by NAA with probe detection | COVID19 positive test | Measurement |
| 723472 | SARS-related coronavirus RNA [Presence] in Unspecified specimen by NAA with probe detection | COVID19 positive test | Measurement |

*For codes in the measurement domain positive was identified using the following codes: 45884084, 45877985, 4181412, 4126681, 9191, 45879438.

### Appendix 2. Code list of study outcomes

| **Concept ID** | **Concept name** |
| --- | --- |
| **Outcome** | **Cerebral venous sinus thrombosis (CVST)** |
| 4102202 | Cerebral venous sinus thrombosis |
| 4048786 | Cerebral venous thrombosis of sigmoid sinus |
| 4043735 | Cerebral venous thrombosis of straight sinus |
| 4111713 | Non-pyogenic venous sinus thrombosis |
| 314667 | Nonpyogenic thrombosis of intracranial venous sinus |
| 4116206 | Septic thrombophlebitis of cavernous sinus |
| 4121335 | Septic thrombophlebitis of lateral sinus |
| 4119136 | Septic thrombophlebitis of sagittal sinus |
| 4041680 | Septic thrombophlebitis of sigmoid sinus |
| 4100225 | Thrombophlebitis lateral venous sinus |
| 4217471 | Thrombophlebitis of basilar sinus |
| 4104695 | Thrombophlebitis of cavernous sinus |
| 4167985 | Thrombophlebitis of inferior sagittal sinus |
| 764714 | Thrombophlebitis of sigmoid sinus |
| 4100224 | Thrombophlebitis of superior longitudinal venous sinus |
| 4098706 | Thrombophlebitis of superior sagittal sinus |
| 4277833 | Thrombophlebitis of torcular Herophili |
| 764710 | Thrombophlebitis of transverse sinus |
| 4228209 | Thrombosis of basilar sinus |
| 4234264 | Thrombosis of cavernous venous sinus |
| 4048890 | Thrombosis of inferior sagittal sinus |
| 4057329 | Thrombosis of lateral venous sinus |
| 4102203 | Thrombosis of superior longitudinal sinus |
| 4290940 | Thrombosis of superior sagittal sinus |
| 4079905 | Thrombosis of torcular Herophili |
| 4105338 | Thrombosis transverse sinus |
| **Outcome** | **DVT** |
| 762047 | Acute bilateral thrombosis of subclavian veins |
| 762148 | Acute deep vein thrombosis of bilateral iliac veins |
| 761444 | Acute deep vein thrombosis of bilateral lower limbs following coronary artery bypass graft |
| 35616028 | Acute deep vein thrombosis of left iliac vein |
| 35615035 | Acute deep vein thrombosis of left lower limb following procedure |
| 761416 | Acute deep vein thrombosis of left upper limb following coronary artery bypass graft |
| 35615031 | Acute deep vein thrombosis of left upper limb following procedure |
| 43531681 | Acute deep vein thrombosis of lower limb |
| 35616027 | Acute deep vein thrombosis of right iliac vein |
| 35615034 | Acute deep vein thrombosis of right lower limb following procedure |
| 761415 | Acute deep vein thrombosis of right upper limb following coronary artery bypass graft |
| 35615030 | Acute deep vein thrombosis of right upper limb following procedure |
| 44782746 | Acute deep venous thrombosis |
| 44782751 | Acute deep venous thrombosis of axillary vein |
| 762008 | Acute deep venous thrombosis of bilateral axillary veins |
| 760875 | Acute deep venous thrombosis of bilateral calves |
| 765155 | Acute deep venous thrombosis of bilateral ileofemoral veins |
| 762017 | Acute deep venous thrombosis of bilateral internal jugular veins |
| 762417 | Acute deep venous thrombosis of bilateral legs |
| 762020 | Acute deep venous thrombosis of bilateral popliteal veins |
| 765546 | Acute deep venous thrombosis of bilateral tibial veins |
| 762004 | Acute deep venous thrombosis of both upper extremities |
| 44782742 | Acute deep venous thrombosis of calf |
| 44782747 | Acute deep venous thrombosis of femoral vein |
| 762015 | Acute deep venous thrombosis of ileofemoral vein of left leg |
| 765541 | Acute deep venous thrombosis of ileofemoral vein of right lower extremity |
| 44782748 | Acute deep venous thrombosis of iliofemoral vein |
| 44782752 | Acute deep venous thrombosis of internal jugular vein |
| 762009 | Acute deep venous thrombosis of left axillary vein |
| 760876 | Acute deep venous thrombosis of left calf |
| 765540 | Acute deep venous thrombosis of left femoral vein |
| 765922 | Acute deep venous thrombosis of left internal jugular vein |
| 762418 | Acute deep venous thrombosis of left lower extremity |
| 765537 | Acute deep venous thrombosis of left upper extremity |
| 44782767 | Acute deep venous thrombosis of lower extremity as complication of procedure |
| 46270071 | Acute deep venous thrombosis of lower limb due to coronary artery bypass grafting |
| 762022 | Acute deep venous thrombosis of politeal vein of right leg |
| 44782743 | Acute deep venous thrombosis of popliteal vein |
| 762021 | Acute deep venous thrombosis of popliteal vein of left leg |
| 762010 | Acute deep venous thrombosis of right axillary vein |
| 760877 | Acute deep venous thrombosis of right calf |
| 762013 | Acute deep venous thrombosis of right femoral vein |
| 762018 | Acute deep venous thrombosis of right internal jugular vein |
| 762419 | Acute deep venous thrombosis of right lower extremity |
| 762005 | Acute deep venous thrombosis of right upper extremity |
| 44782745 | Acute deep venous thrombosis of thigh |
| 44782744 | Acute deep venous thrombosis of tibial vein |
| 762026 | Acute deep venous thrombosis of tibial vein of left leg |
| 765156 | Acute deep venous thrombosis of tibial vein of right leg |
| 44782421 | Acute deep venous thrombosis of upper extremity |
| 764016 | Acute deep venous thrombosis of upper extremity after coronary artery bypass graft |
| 44782766 | Acute deep venous thrombosis of upper extremity as complication of procedure |
| 762048 | Acute thrombosis of left subclavian vein |
| 45757410 | Acute thrombosis of mesenteric vein |
| 762049 | Acute thrombosis of right subclavian vein |
| 36712892 | Acute thrombosis of splenic vein |
| 44782762 | Acute thrombosis of subclavian vein |
| 37109253 | Bilateral acute deep vein thrombosis of femoral veins |
| 40478951 | Bilateral deep vein thrombosis of lower extremities |
| 4046884 | Deep vein thrombosis of leg related to air travel |
| 4133004 | Deep venous thrombosis |
| 4181315 | Deep venous thrombosis associated with coronary artery bypass graft |
| 45773536 | Deep venous thrombosis of femoropopliteal vein |
| 763942 | Deep venous thrombosis of left lower extremity |
| 761980 | Deep venous thrombosis of left upper extremity |
| 443537 | Deep venous thrombosis of lower extremity |
| 4133975 | Deep venous thrombosis of pelvic vein |
| 40480555 | Deep venous thrombosis of peroneal vein |
| 4322565 | Deep venous thrombosis of profunda femoris vein |
| 763941 | Deep venous thrombosis of right lower extremity |
| 761928 | Deep venous thrombosis of right upper extremity |
| 4207899 | Deep venous thrombosis of tibial vein |
| 4028057 | Deep venous thrombosis of upper extremity |
| 193512 | Embolism and thrombosis of the renal vein |
| 435565 | Embolism and thrombosis of the vena cava |
| 4119760 | Iliofemoral deep vein thrombosis |
| 4124856 | Inferior mesenteric vein thrombosis |
| 4281689 | Phlegmasia alba dolens |
| 4284538 | Phlegmasia cerulea dolens |
| 4309333 | Postoperative deep vein thrombosis |
| 46285905 | Provoked deep vein thrombosis |
| 4033521 | Splenic vein thrombosis |
| 4055089 | Superior mesenteric vein thrombosis |
| 42538533 | Thrombosis of iliac vein |
| 44811347 | Thrombosis of internal jugular vein |
| 765049 | Thrombosis of left peroneal vein |
| 4317289 | Thrombosis of mesenteric vein |
| 4203836 | Thrombosis of subclavian vein |
| 4175649 | Thrombosis of the popliteal vein |
| 4153353 | Traumatic thrombosis of axillary vein |
| 46285904 | Unprovoked deep vein thrombosis |
| 4221821 | Thrombophlebitis of deep veins of lower extremity |
| 46271900 | Recurrent deep vein thrombosis |
| 4189004 | Deep vein thrombosis of leg related to intravenous drug use |
| **Outcome** | **SVT** |
| 4033521 | Splenic vein thrombosis |
| 36712892 | Acute thrombosis of splenic vein |
| 4033521 | Splenic vein thrombosis |
| 196715 | Budd-Chiari syndrome |
| 199837 | Portal vein thrombosis |
| 4317289 | Thrombosis of mesenteric vein |
| 4092406 | Portal thrombophlebitis |
| 36712892 | Acute thrombosis of splenic vein |
| 4173167 | Mesenteric embolus |
| 4144032 | Mesenteric thrombus and/or embolus |
| 45757410 | Acute thrombosis of mesenteric vein |
| 45757409 | Chronic thrombosis of mesenteric vein |
| 4318407 | Thrombophlebitis of mesenteric vein |
| 4124856 | Inferior mesenteric vein thrombosis |
| 4055089 | Superior mesenteric vein thrombosis |
| 199837 | Portal vein thrombosis |
| 36717492 | Acute occlusion of mesenteric vein |
| 45757410 | Acute thrombosis of mesenteric vein |
| 4124856 | Inferior mesenteric vein thrombosis |
| 4055089 | Superior mesenteric vein thrombosis |
| 4317289 | Thrombosis of mesenteric vein |
| 45757409 | Chronic thrombosis of mesenteric vein |
| 4318407 | Thrombophlebitis of mesenteric vein |
| 4173167 | Mesenteric embolus |
| 4144032 | Mesenteric thrombus and/or embolus |
| 36717492 | Acute occlusion of mesenteric vein |
| 36712892 | Acute thrombosis of splenic vein |
| 196715 | Budd-Chiari syndrome |
| 35624285 | Complete obstruction of hepatic portal vein |
| 4301208 | Hepatic vein thrombosis |
| 37110194 | Hepatic veno-occlusive disease with immunodeficiency syndrome |
| 37109927 | Obstruction of visceral vein |
| 4238060 | Portal vein obstruction |
| 4033521 | Splenic vein thrombosis |
| 4277276 | Veno-occlusive disease of the liver |
| 37111372 | Visceral venous thrombosis |
| 36712891 | Chronic thrombosis of splenic vein |
| **Outcome** | **Pulmonary embolism** |
| 4120091 | Acute massive pulmonary embolism |
| 45768439 | Acute pulmonary embolism |
| 45768888 | Acute pulmonary thromboembolism |
| 4309039 | Hemorrhagic pulmonary infarction |
| 762808 | Infarction of lung due to embolus |
| 40480461 | Infarction of lung due to iatrogenic pulmonary embolism |
| 4108681 | Postoperative pulmonary embolus |
| 4091708 | Pulmonary air embolism |
| 440417 | Pulmonary embolism |
| 37109911 | Pulmonary embolism due to and following acute myocardial infarction |
| 37016922 | Pulmonary embolism on long-term anticoagulation therapy |
| 43530605 | Pulmonary embolism with pulmonary infarction |
| 4119608 | Pulmonary fat embolism |
| 254662 | Pulmonary infarction |
| 4253796 | Pulmonary microemboli |
| 45766471 | Pulmonary oil microembolism |
| 4121618 | Pulmonary thromboembolism |
| 4119610 | Pulmonary tumor embolism |
| 4119607 | Subacute massive pulmonary embolism |
| 4119609 | Subacute pulmonary fat embolism |
| 4236271 | Recurrent pulmonary embolism |
| **Outcome** | **Myocardial infarction** |
| 4119457 | Acute Q wave infarction - anterolateral |
| 4119943 | Acute Q wave infarction - anteroseptal |
| 4121464 | Acute Q wave infarction - inferior |
| 4121465 | Acute Q wave infarction - inferolateral |
| 4124684 | Acute Q wave infarction - lateral |
| 4119948 | Acute Q wave infarction - widespread |
| 4126801 | Acute Q wave myocardial infarction |
| 4296653 | Acute ST segment elevation myocardial infarction |
| 46270162 | Acute ST segment elevation myocardial infarction due to left coronary artery occlusion |
| 761737 | Acute ST segment elevation myocardial infarction due to occlusion of circumflex coronary artery |
| 46270163 | Acute ST segment elevation myocardial infarction due to right coronary artery occlusion |
| 43020460 | Acute ST segment elevation myocardial infarction involving left anterior descending coronary artery |
| 45766076 | Acute ST segment elevation myocardial infarction of anterior wall involving right ventricle |
| 761736 | Acute ST segment elevation myocardial infarction of anteroapical wall |
| 46270159 | Acute ST segment elevation myocardial infarction of anterolateral wall |
| 46270160 | Acute ST segment elevation myocardial infarction of anteroseptal wall |
| 45766116 | Acute ST segment elevation myocardial infarction of inferior wall |
| 45766151 | Acute ST segment elevation myocardial infarction of inferior wall involving right ventricle |
| 35611570 | Acute ST segment elevation myocardial infarction of inferolateral wall |
| 35611571 | Acute ST segment elevation myocardial infarction of inferoposterior wall |
| 46274044 | Acute ST segment elevation myocardial infarction of lateral wall |
| 46270161 | Acute ST segment elevation myocardial infarction of posterior wall |
| 46273495 | Acute ST segment elevation myocardial infarction of posterobasal wall |
| 46270158 | Acute ST segment elevation myocardial infarction of posterolateral wall |
| 46270164 | Acute ST segment elevation myocardial infarction of septum |
| 45766075 | Acute anterior ST segment elevation myocardial infarction |
| 4178129 | Acute anteroapical myocardial infarction |
| 4267568 | Acute anteroseptal myocardial infarction |
| 312327 | Acute myocardial infarction |
| 44782769 | Acute myocardial infarction due to left coronary artery occlusion |
| 44782712 | Acute myocardial infarction due to right coronary artery occlusion |
| 45766115 | Acute myocardial infarction during procedure |
| 434376 | Acute myocardial infarction of anterior wall |
| 45766150 | Acute myocardial infarction of anterior wall involving right ventricle |
| 438438 | Acute myocardial infarction of anterolateral wall |
| 4243372 | Acute myocardial infarction of apical-lateral wall |
| 4108669 | Acute myocardial infarction of atrium |
| 4151046 | Acute myocardial infarction of basal-lateral wall |
| 4275436 | Acute myocardial infarction of high lateral wall |
| 438170 | Acute myocardial infarction of inferior wall |
| 45771322 | Acute myocardial infarction of inferior wall involving right ventricle |
| 438447 | Acute myocardial infarction of inferolateral wall |
| 441579 | Acute myocardial infarction of inferoposterior wall |
| 436706 | Acute myocardial infarction of lateral wall |
| 4324413 | Acute myocardial infarction of posterobasal wall |
| 4051874 | Acute myocardial infarction of posterolateral wall |
| 4303359 | Acute myocardial infarction of septum |
| 4147223 | Acute myocardial infarction with rupture of ventricle |
| 4145721 | Acute non-Q wave infarction |
| 4119944 | Acute non-Q wave infarction - anterolateral |
| 4119456 | Acute non-Q wave infarction - anteroseptal |
| 4119945 | Acute non-Q wave infarction - inferior |
| 4119946 | Acute non-Q wave infarction - inferolateral |
| 4121466 | Acute non-Q wave infarction - lateral |
| 4124685 | Acute non-Q wave infarction - widespread |
| 4270024 | Acute non-ST segment elevation myocardial infarction |
| 35610091 | Acute nontransmural myocardial infarction |
| 319039 | Acute posterior myocardial infarction |
| 444406 | Acute subendocardial infarction |
| 35610093 | Acute transmural myocardial infarction |
| 4119947 | Acute widespread myocardial infarction |
| 37109912 | Arrhythmia due to and following acute myocardial infarction |
| 438172 | Atrial septal defect due to and following acute myocardial infarction |
| 4124687 | Cardiac rupture due to and following acute myocardial infarction |
| 4215259 | First myocardial infarction |
| 4108678 | Hemopericardium due to and following acute myocardial infarction |
| 4173632 | Microinfarct of heart |
| 45771327 | Mitral valve regurgitation due to acute myocardial infarction with papillary muscle and chordal rupture |
| 45766214 | Mitral valve regurgitation due to acute myocardial infarction without papillary muscle and chordal rupture |
| 45766212 | Mitral valve regurgitation due to and following acute myocardial infarction |
| 4323202 | Mixed myocardial ischemia and infarction |
| 4329847 | Myocardial infarction |
| 37309626 | Myocardial infarction due to demand ischemia |
| 4170094 | Myocardial infarction in recovery phase |
| 4200113 | Non-Q wave myocardial infarction |
| 4030582 | Postoperative myocardial infarction |
| 35610087 | Postoperative nontransmural myocardial infarction |
| 4206867 | Postoperative subendocardial myocardial infarction |
| 35610089 | Postoperative transmural myocardial infarction |
| 4207921 | Postoperative transmural myocardial infarction of anterior wall |
| 4209541 | Postoperative transmural myocardial infarction of inferior wall |
| 37109911 | Pulmonary embolism due to and following acute myocardial infarction |
| 4108679 | Rupture of cardiac wall without hemopericardium as current complication following acute myocardial infarction |
| 4108219 | Rupture of chordae tendinae due to and following acute myocardial infarction |
| 4124686 | Silent myocardial infarction |
| 765132 | Subendocardial myocardial infarction |
| 45766114 | Subsequent ST segment elevation myocardial infarction |
| 45766113 | Subsequent ST segment elevation myocardial infarction of anterior wall |
| 45773170 | Subsequent ST segment elevation myocardial infarction of inferior wall |
| 4108217 | Subsequent myocardial infarction |
| 4108677 | Subsequent myocardial infarction of anterior wall |
| 4108218 | Subsequent myocardial infarction of inferior wall |
| 45766241 | Subsequent non-ST segment elevation myocardial infarction |
| 4108680 | Thrombosis of atrium, auricular appendage, and ventricle due to and following acute myocardial infarction |
| 439693 | True posterior myocardial infarction |
| 37109910 | Ventricular aneurysm due to and following acute myocardial infarction |
| **Outcome** | **Heart failure** |
| 44782718 | Acute combined systolic and diastolic heart failure |
| 4023479 | Acute congestive heart failure |
| 312927 | Acute cor pulmonale |
| 40481042 | Acute diastolic heart failure |
| 44782655 | Acute exacerbation of chronic congestive heart failure |
| 442310 | Acute heart failure |
| 764877 | Acute heart failure co-occurrent with normal ejection fraction |
| 4108245 | Acute left ventricular failure |
| 4327205 | Acute left-sided congestive heart failure |
| 4267800 | Acute left-sided heart failure |
| 44782733 | Acute on chronic combined systolic and diastolic heart failure |
| 40481043 | Acute on chronic diastolic heart failure |
| 764874 | Acute on chronic heart failure co-occurrent with normal ejection fraction |
| 37309625 | Acute on chronic right-sided congestive heart failure |
| 40480602 | Acute on chronic systolic heart failure |
| 4215446 | Acute right-sided congestive heart failure |
| 4233424 | Acute right-sided heart failure |
| 40480603 | Acute systolic heart failure |
| 4193236 | Ayerza's syndrome |
| 439698 | Benign hypertensive heart disease with congestive cardiac failure |
| 4030258 | Bernheim's syndrome |
| 4242669 | Biventricular congestive heart failure |
| 4215802 | Cardiac asthma |
| 4177493 | Cardiac insufficiency due to prosthesis |
| 4233224 | Cardiac insufficiency during AND/OR resulting from a procedure |
| 4264636 | Cardiac insufficiency following cardiac surgery |
| 4259490 | Cardiorespiratory failure |
| 44782719 | Chronic combined systolic and diastolic heart failure |
| 4229440 | Chronic congestive heart failure |
| 4195892 | Chronic cor pulmonale |
| 40479576 | Chronic diastolic heart failure |
| 444031 | Chronic heart failure |
| 764876 | Chronic heart failure co-occurrent with normal ejection fraction |
| 4206009 | Chronic left-sided congestive heart failure |
| 4009047 | Chronic left-sided heart failure |
| 4284562 | Chronic right-sided congestive heart failure |
| 4014159 | Chronic right-sided heart failure |
| 40479192 | Chronic systolic heart failure |
| 4108244 | Compensated cardiac failure |
| 319835 | Congestive heart failure |
| 44784345 | Congestive heart failure as early postoperative complication |
| 762002 | Congestive heart failure as post-operative complication of cardiac surgery |
| 762003 | Congestive heart failure as post-operative complication of non-cardiac surgery |
| 44782428 | Congestive heart failure due to cardiomyopathy |
| 4139864 | Congestive heart failure due to left ventricular systolic dysfunction |
| 4142561 | Congestive heart failure due to valvular disease |
| 36713488 | Congestive heart failure stage B |
| 36712928 | Congestive heart failure stage B due to ischemic cardiomyopathy |
| 43021826 | Congestive heart failure stage C |
| 36712927 | Congestive heart failure stage C due to ischemic cardiomyopathy |
| 43021825 | Congestive heart failure stage D |
| 44782713 | Congestive heart failure with right heart failure |
| 4307356 | Cor pulmonale |
| 4111554 | Decompensated cardiac failure |
| 4311437 | Decompensated chronic heart failure |
| 443587 | Diastolic heart failure |
| 43530643 | Diastolic heart failure stage B |
| 43021842 | Diastolic heart failure stage C |
| 43021841 | Diastolic heart failure stage D |
| 43022068 | Exacerbation of congestive heart failure |
| 316139 | Heart failure |
| 4124705 | Heart failure as a complication of care |
| 37311948 | Heart failure with mid range ejection fraction |
| 40486933 | Heart failure with normal ejection fraction |
| 45766164 | Heart failure with reduced ejection fraction |
| 45766167 | Heart failure with reduced ejection fraction due to cardiomyopathy |
| 45766165 | Heart failure with reduced ejection fraction due to coronary artery disease |
| 45773075 | Heart failure with reduced ejection fraction due to heart valve disease |
| 45766166 | Heart failure with reduced ejection fraction due to myocarditis |
| 4004279 | High output heart failure |
| 44782728 | Hypertensive heart AND chronic kidney disease with congestive heart failure |
| 439696 | Hypertensive heart and renal disease with (congestive) heart failure |
| 439694 | Hypertensive heart and renal disease with both (congestive) heart failure and renal failure |
| 314378 | Hypertensive heart disease with congestive heart failure |
| 444101 | Hypertensive heart failure |
| 439846 | Left heart failure |
| 4185565 | Low cardiac output syndrome |
| 4103448 | Low output heart failure |
| 316994 | Malignant hypertensive heart disease with congestive heart failure |
| 4141124 | Postvalvulotomy syndrome |
| 764873 | Reduced ejection fraction co-occurrent and due to acute heart failure |
| 764871 | Reduced ejection fraction co-occurrent and due to acute on chronic heart failure |
| 764872 | Reduced ejection fraction co-occurrent and due to chronic heart failure |
| 4199500 | Refractory heart failure |
| 4138307 | Right heart failure due to pulmonary hypertension |
| 4195785 | Right heart failure secondary to left heart failure |
| 4273632 | Right ventricular failure |
| 35615055 | Saddle embolus of pulmonary artery with acute cor pulmonale |
| 4079695 | Sepsis-associated left ventricular failure |
| 4079296 | Sepsis-associated right ventricular failure |
| 44784442 | Symptomatic congestive heart failure |
| 443580 | Systolic heart failure |
| 43530642 | Systolic heart failure stage B |
| 36717359 | Systolic heart failure stage B due to ischemic cardiomyopathy |
| 43020421 | Systolic heart failure stage C |
| 36712929 | Systolic heart failure stage C due to ischemic cardiomyopathy |
| 43021840 | Systolic heart failure stage D |
| 40482857 | Cardiorenal syndrome |
| 4153875 | Cardiac insufficiency as a complication of care |
| 4215511 | Emergency hospital admission for heart failure |
| 4215689 | Heart failure confirmed |
| 4173819 | Impaired left ventricular function |

### Appendix 3. Definition of immunocompromised status at the index date

Individuals who are immunocompromised at the index data were defined by the recording of certain conditions or certain conditions plus treatments prior to index date. Individuals were considered immunocompromised if they have one or more of the following conditions recorded within 365 days prior to index date:

- HIV/AIDS
- Haematological malignancies
- Solid malignancies
- Other intrinsic immune conditions

Individuals were defined as being immunocompromised if they were treated with antineoplastic and immunomodulating agents between 183 days to one day prior to index date.

Individuals were also defined as being immunocompromised if they were treated with systemic corticosteroids between 183 days to one day prior to index date and had a recording of the following within 365 days prior to index date:

- Organ transplantations
- Rheumatologic/inflammatory conditions (rheumatoid arthritis, inflammatory bowel disease, and systemic lupus erythematosus)

### Appendix 3. Supplement tables and figures

**Figure S1:** Age-sex specific incidence rates of study events among the background population.


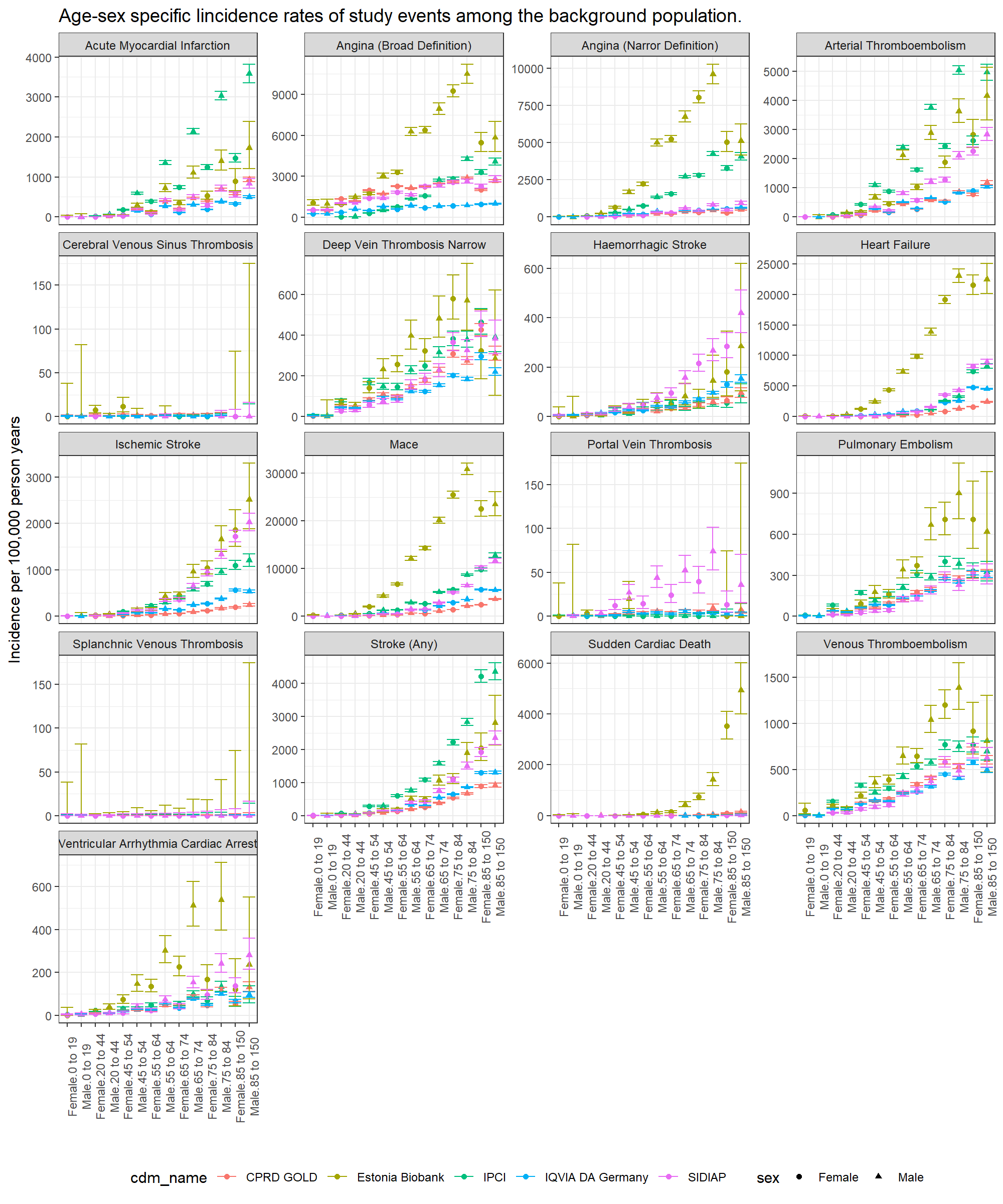


**Figure S2.** Crude incidence rates after infection, stratified by Covid-19 infection history.

| 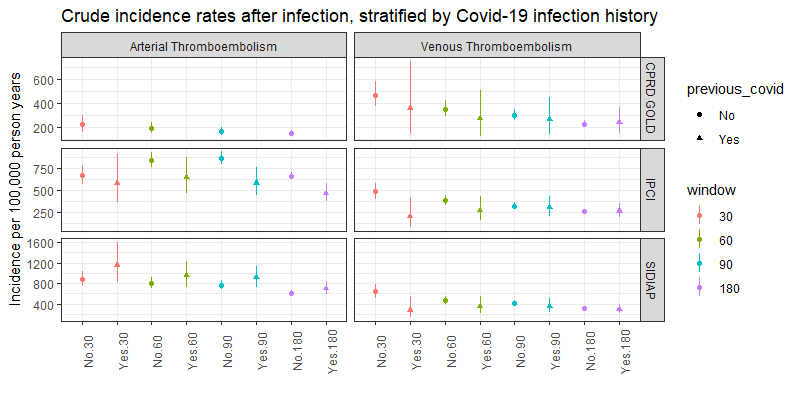 |
| --- |

* number of events among people with Covid-19 history in CPRD GOLD was below 5 thus not shown here.

**Figure S3.** Incidence rates after infection, stratified by number of doses of vaccines received prior to infection.


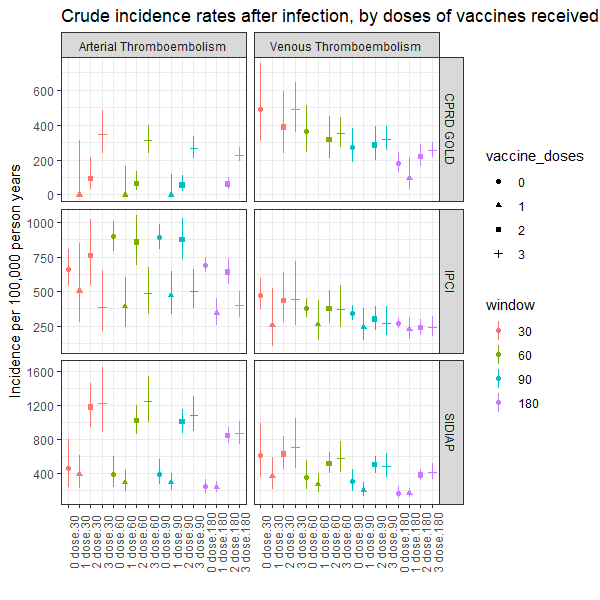


* number of events among people with Covid-19 history in CPRD GOLD was below 5 thus not shown here.

**Figure S4.** Age-sex specific incidence rate ratios of ATE among people with COVID-19 infection stratified by age and sex, by database.


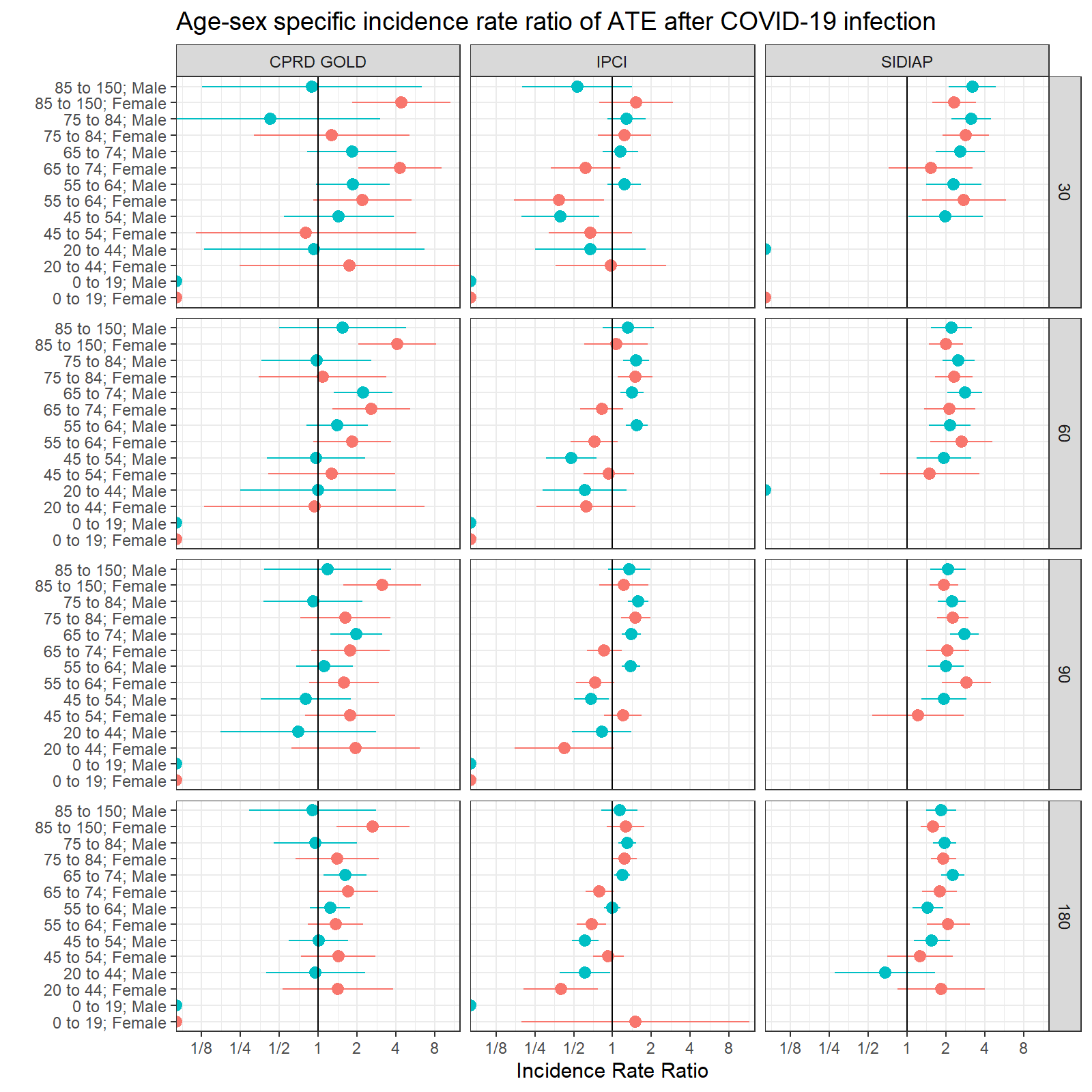


**Figure S5.** Age-sex specific incidence rate ratios of VTE among people with COVID-19 infection stratified by age and sex, by database.


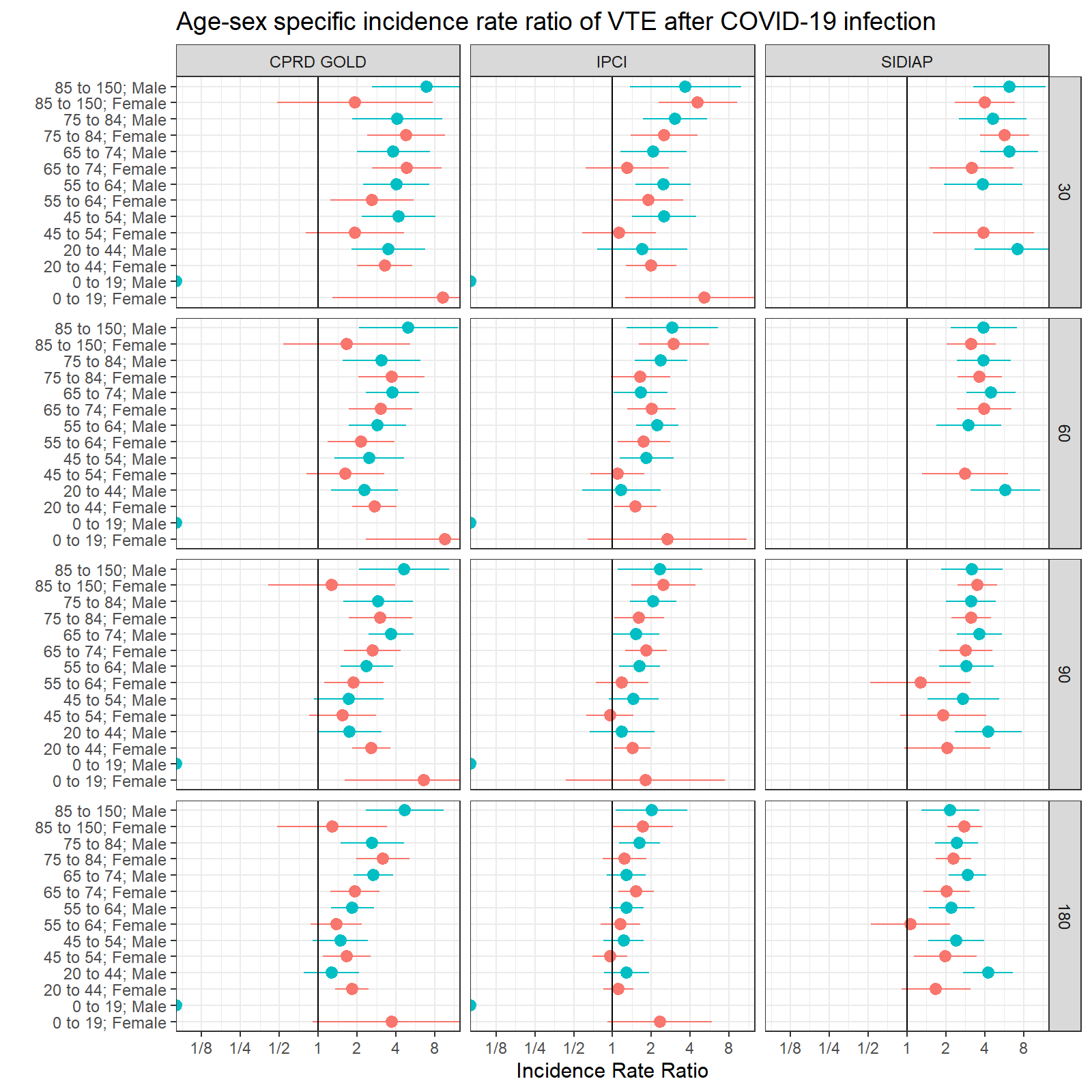


**S6. Sensitivity analysis, Standardised incidence rate ratio of ATE and VTE stratified by vaccine and covid status, and immunocompromised status.**


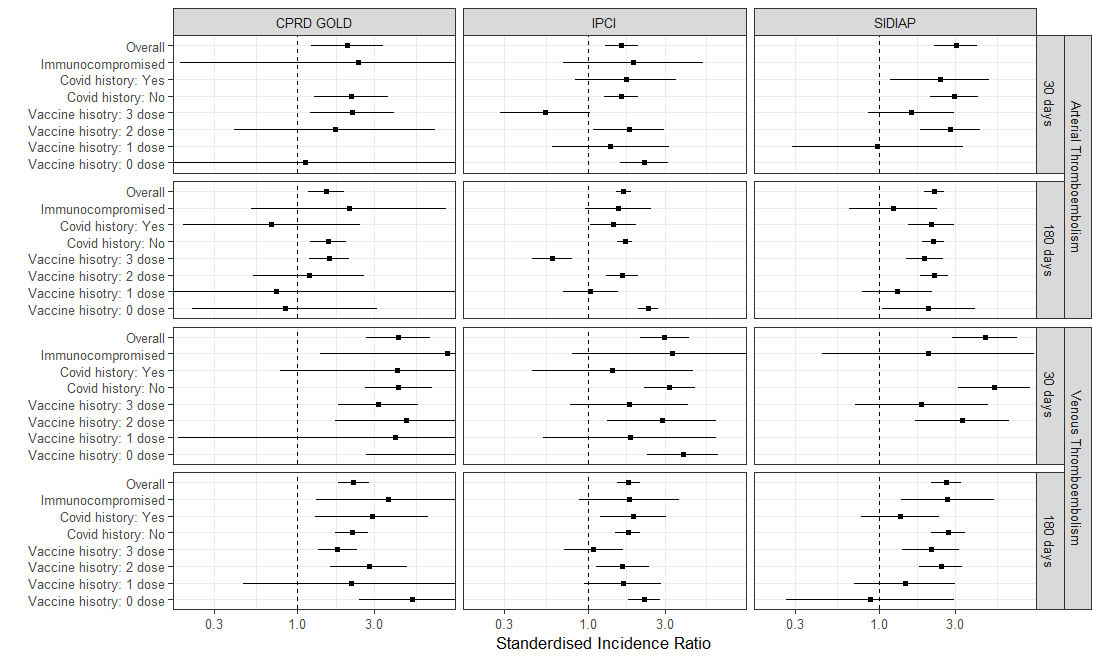

Supplement: Supplementary file 1 — Supplementary Material 1. [file 41598_2026_51445_MOESM1_ESM.docx]
